# Assessing total cost of driving competitiveness of zero-emission trucks

**DOI:** 10.1016/j.isci.2024.109385

**Published:** 2024-03-02

**Authors:** Catherine Ledna, Matteo Muratori, Arthur Yip, Paige Jadun, Christopher Hoehne, Kara Podkaminer

**Affiliations:** 1 National Renewable Energy Laboratory, Golden, CO, USA; 2 US Department of Energy, Washington, DC, USA

**Keywords:** Energy resources, Energy policy, Engineering

## Abstract

Medium- and heavy-duty vehicles are 21% of US transportation greenhouse gas (GHG) emissions and a major source of air pollution. We explore how the total cost of driving (TCD) of zero-emission vehicles (ZEVs), including battery electric vehicles and hydrogen fuel cell electric vehicles (EVs and FCEVs), could evolve under alternative scenarios. With continued improvements in vehicles and fuels, ZEVs can rapidly become viable, potentially reaching TCD parity or better compared to diesel vehicles by 2035 for all market segments. For heavy long-haul trucks, EVs become competitive on a TCD basis at charging costs below $0.18/kWh, while FCEVs become competitive on a TCD basis at hydrogen costs below $5/kg. A full transition to ZEV sales by 2035 results in 65% emissions reductions by 2050 compared to 2019 without supportive policies. Incentives such as the Inflation Reduction Act vehicle purchase credits further accelerate ZEV TCD competitiveness with major adoption opportunities over the next five years.

## Highlights

• We model the total cost of driving for all trucks (Classes 3–8) in the US
• ZEVs can reach total cost of driving parity by 2035 in all market segments
• 2050 GHG emissions can be reduced by 65% from 2019 levels under central assumptions
• Sensitivities explore tax credits, fuel costs, and technology progress assumptions

## Graphical abstract

**Figure:**
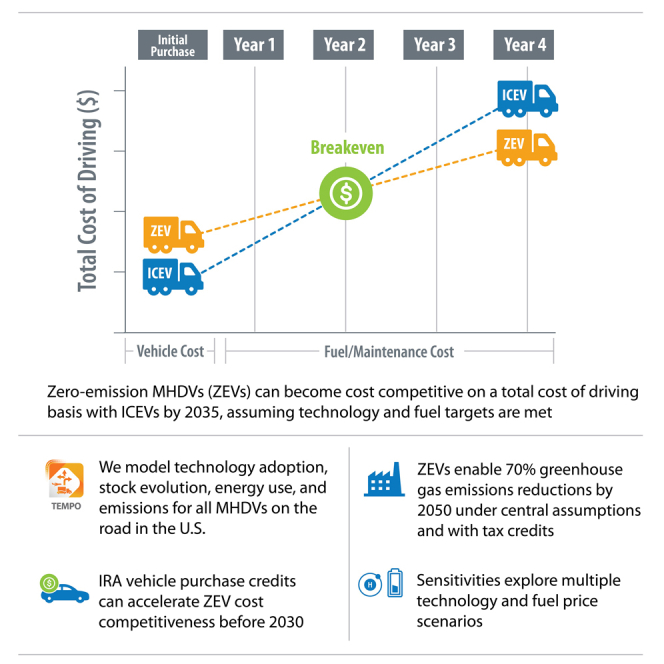

## Introduction

Medium and heavy-duty vehicles (MHDVs) – on-road vehicles with a gross vehicle weight rating (GVWR) of over 10,000 pounds used for a wide variety of applications – account for 5% of vehicles on the road in the United States, but are responsible for 21% of US transportation greenhouse gas (GHG) emissions, with future growth expected to outpace that of light-duty vehicles.^1^^,^^2^^,^^3^ Reducing MHDV emissions is a key priority for both climate change mitigation and efforts to improve local air quality. Zero-emission vehicles (ZEVs), which include vehicles with no tailpipe emissions such as battery electric vehicles (EVs) and hydrogen fuel cell electric vehicles (FCEVs), have been identified as key solutions to achieve these goals.^1^^,^^4^^,^^5^ Proposed and announced policy actions include strengthened air quality and GHG emissions standards, ZEV purchase subsidies, investments in research and development, and infrastructure deployments.^6^^,^^7^ For example, the Inflation Reduction Act (IRA) includes tax credits of up to $40,000 for qualifying clean vehicle purchases, including EVs and FCEVs as well as incentives for refueling infrastructure.^8^ More recently, the US Environmental Protection Agency has proposed more stringent GHG emission rules for MHDVs for model years 2027–2032.^9^ We present a comprehensive model to estimate how the total cost of driving of zero-emission EVs and FCEVs could evolve under alternative scenarios of future technology progress and fuel costs. We use these insights to explore how ZEV adoption could evolve for all vehicle classes, compute fleet turnover and energy consumption, and evaluate multiple technology, fuel, and policy sensitivities to inform the discourse around all MHDV zero emission vehicles.

**Figure 1:**
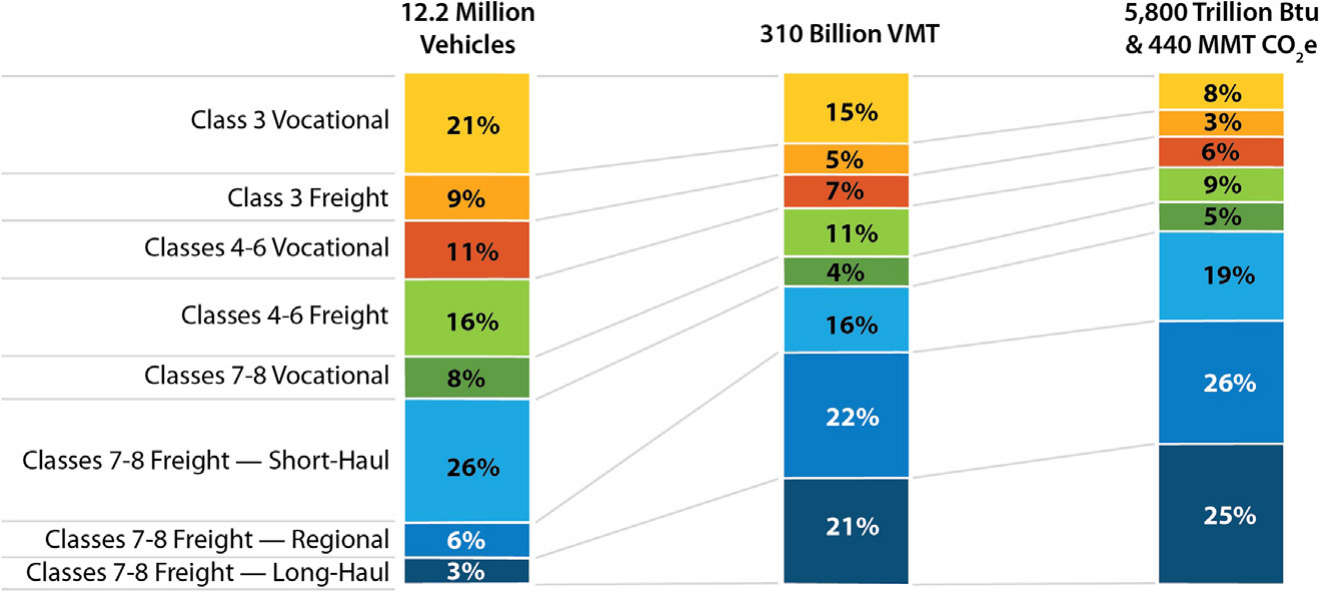
Comparing 2019 MHDV Vehicle Stock, VMT, Energy Use and GHG Emissions Shares by Vehicle Class and Application Based on data from the Annual Energy Outlook, the Freight Analysis Framework, and the 2002 Vehicle Inventory and Use. Survey.^16^^,^^17^^,^^18^ Colors distinguish different vehicle classes, while shades refer to operational segments. Lighter shades refer to shorter-distance operational segments. Larger vehicles (heavy Class 7–8 trucks) tend to be driven more and have lower fuel economies, resulting in disproportionate energy needs and GHG emissions.

To date, ZEVs remain a small share of existing MHDVs (approximately 3,100 vehicles on the road in the US as of 2022, excluding buses^10^) and face near-term barriers such as high purchase costs, a lack of charging or refueling infrastructure, and logistical challenges to fleet conversion.^11^^,^^12^ However, with recent technology progress and investments in solutions to decarbonize transportation spurred by public and private interest, EVs are rapidly becoming a viable solution.^10^^,^^13^^,^^14^ FCEVs are not yet competitive in MHDV applications even when considering vehicle purchase credits but could provide cost-effective solutions for harder-to-electrify market segments such as long-haul and multi-shift applications.^15^ There are many different market segments for MHDVs with different technical and economic requirements, resulting in differences in the cost competitiveness of zero-emission technologies. The US MHDV fleet encompasses vehicles ranging from 10,000 pounds GVWR to over 33,000 pounds, driving distances as low as under 10,000 miles per year to above 200,000 miles per year, and serving a variety of freight and non-freight applications. Figure 1 summarizes the distribution of vehicle stock, vehicle-miles traveled (VMT), and energy and GHG emissions across the US MHDV fleet in 2019, considering all freight and non-freight MHDVs with a GVWR of over 10,000 pounds except buses.^3^^,^^16^^,^^17^^,^^18^ Heavy trucks (Classes 7–8, with a GVWR of 26,000 pounds or greater) account for 43% of the total MHDV stock but are responsible for 63% of VMT and 74% of energy use and GHG emissions, with long-haul trucks contributing a disproportionate share of emissions. Medium-duty vehicles (Classes 3–6, or GVWR of 10,000 to 26,000 pounds) tend to be driven less and are more fuel efficient, resulting in emissions that are disproportionately lower than their share of stock. The substantial differences between stock and emissions shares suggest that high ZEV sales or stock shares may not translate into proportional GHG emissions reductions, depending on vehicle size and annual use. The heterogeneity of the MHDV fleet also has implications for ZEV competitiveness with internal combustion engine vehicles (ICEVs) and hybrid electric vehicles (HEVs) on a total cost of driving basis, implying different requirements and opportunities to optimize vehicle design (including vehicle range, power and weight requirements) and infrastructure needs (slow or fast charging for EVs and local or regional refueling networks for EVs and FCEVs).

While projecting future technology adoption is complex and many factors influence consumer decisions, economics is a key determinant of technology choices for commercial applications. Past studies have evaluated the competitiveness of zero-emission technologies for selected vehicle classes and applications in US and international settings, frequently using total cost of ownership (TCO) or total cost of driving (TCD) metrics. Both metrics consider upfront vehicle costs and recurring operational costs (including fuel and maintenance costs) discounted over a period of time, typically shorter than the ownership period or lifetime of the vehicle (i.e., comparing financial performance over the first few years of vehicle operation). TCO may additionally consider operational costs such as driver wages, insurance, and resale or salvage value omitted by TCD. Other costs such as the cost of payload limitations or time spent refueling may be incorporated into these frameworks depending on the study. While cost analyses in general are highly sensitive to input assumptions, including uncertain projections of future technology and fuel costs, multiple studies have found that ZEV technologies can become cost competitive on a TCO or TCD basis with diesel vehicles in some or all classes and market segments between 2030 and 2040. Hunter et al.^15^ estimate TCO for conventional, hybrid, and ZEV technologies for medium and heavy trucks in the US, finding that EVs can become competitive with conventional and hybrid technologies before 2050 for single-shift operations without weight limitations, while FCEVs can become competitive prior to 2050 in multi-shift, weight-limited operations. Analysis by ITF^19^ and Tanco et al.^20^ evaluate ZEV cost competitiveness on a TCO basis in European and Latin American settings respectively, finding TCO competitiveness between 2030 and 2050 across multiple vehicle classes and market segments, particularly for lighter short-haul vehicles. Other analyses have found that TCO competitiveness can be reached on earlier timescales. Both Phadke et al.^21^ and the ICCT^22^^,^^23^ find that U.S. EVs can achieve TCO parity with conventional technologies at or before 2030 for Class 8 long-haul applications; Moultak et al.^23^ also find that FCEVs can achieve parity on this timescale. Burke et al.^24^ and find that EVs and FCEVs can approach TCO parity with ICEVs by 2030 in long and short-haul applications. In general, these analyses suggest that ZEVs in lighter vehicle classes are likely to achieve TCO or TCD competitiveness with conventional and hybrid technologies earlier than heavier classes (before 2030), with EVs being the most competitive technology in these classes. FCEV competitiveness has been found to be highly contingent on the cost of hydrogen and on assumptions about competing EV technologies. Hunter et al.^15^ suggest that fuel cells have greater opportunities in market segments that have high opportunity costs from time spent recharging (e.g., long-haul operations). Other studies evaluate electrification potential based on fleet operational characteristics without considering cost competitiveness. Lund et al.^25^ find that electrification is feasible in the present day for a substantial portion of MHDV fleets in California and New York (65% of medium-duty and 50% of heavy-duty trucks), based on an analysis of existing duty cycles. Liimatainen et al.^26^ and Cabukoglu et al.^27^ also evaluate electrification potential of the MHDV fleet in a European context. Their findings suggest that near-term electrification potential may be limited by weight restrictions and high daily travel distances but may be high particularly for medium-duty trucks. Other studies in European contexts such as Earl et al.^28^ and Ainalis et al.^29^ suggest that EVs are suitable for long-haul applications and may be more competitive than FCEVs due to their higher efficiency and greater fuel cost and maintenance savings.

While many recent studies have evaluated the economic competitiveness and technical feasibility of ZEVs in one or more market segments, few studies to date consider vehicle TCD competitiveness, adoption, energy consumption, and fleet turnover in a holistic framework across all MHDV applications. Moultak et al.^23^ evaluate the energy and emissions implications of varying EV and FCEV penetration rates in the US, Europe, and China using a fleet turnover model, but use exogenously determined adoption trajectories rather than endogenously computing them based on cost or other factors. Ledna et al.^3^ present an initial holistic framework for the US MHDV fleet, finding that ZEVs can achieve TCD parity by 2035 in all market segments and that substantial emissions reductions are achievable without accelerated fleet turnover under central assumptions. This study builds on Ledna et al. by updating its assumptions, including additional sensitivities, and expanding its analysis to include the impacts of vehicle purchase credits in the 2022 IRA. In this study, we evaluate ZEV market penetration, energy consumption, and emissions reduction potential for all US MHDV market segments from the present day to 2050 from an economic perspective. Our analysis captures US-specific operational characteristics based on a synthesis of the 2002 Vehicle Inventory and Use Survey (VIUS)^18^ and the Freight Analysis Framework (FAF),^17^ including differences in daily and annual VMT across segments. We use the Transportation Energy & Mobility Pathway Options (TEMPO) model,^30^ a sector-wide transportation energy systems model, to estimate the total cost of driving of all MHDV technologies, as well as new vehicle purchases, stock turnover, vehicle activity, and resulting energy consumption and GHG emissions. Our analysis uniquely captures differences across various MHDV market segments in vehicle use and energy consumption. We consider a range of scenarios, encompassing technology cost and progress sensitivities, fuel costs, and policy, including the impact of the 2022 IRA MHDV commercial vehicle tax credits (Provision 45W), highlighting its impact on TCD competitiveness. Other factors such as infrastructure or manufacturing constraints, which could lower or delay real-world ZEV sales, are not addressed here. Our analysis aims to answer the following questions.

(1) Based on technology and fuel progress trajectories developed by the US Department of Energy (DOE) (described further in the STAR Methods section), what is the near-term (2030) and long-term (2050) ZEV adoption potential for MHDVs?
(2) How do sensitivities surrounding technology and fuel progress affect ZEV adoption and emissions reductions?
(3) How do ZEV adoption potential and emissions reductions vary across MHDV market segments?
(4) How might IRA Provision 45W tax credits affect near-term MHDV TCD competitiveness and potential emissions reductions?

Our findings suggest that with continued progress in vehicle and fuel technologies – aligned with US DOE and industry-vetted targets – ZEVs can reach parity with conventional and hybrid diesel technologies by 2035 across all US market segments. We find substantial variation across technology and fuel sensitivities, particularly around EV and FCEV tradeoffs. Under central assumptions and absent IRA tax credits, ZEVs are the lowest cost technology for 38% of all MHDV sales by 2030 as a result of lower combined vehicle purchase and operating costs over the first few years of vehicle operation. IRA vehicle purchase incentives can greatly increase the share of sales for which ZEV is cheaper than conventional vehicles (to over 80% by 2030, assuming no manufacturing or infrastructure constraints). Overall emissions reductions under central assumptions are roughly 65% by 2050 relative to 2019 levels, with limitations due to slow fleet stock turnover, and roughly 70% with IRA purchase credits. Emissions implications also vary substantially in sensitivities.

## Results

### Central scenario findings

We first evaluate the TCD competitiveness of conventional, hybrid and zero-emission MHDV technologies under central assumptions (see Table 1). Central technology assumptions are based on the US DOE’s high technology progress scenarios for EV and FCEV technologies (as documented in Islam et al.^31^) and are vetted with industry and exclude supportive policies such as the IRA. We also consider 10 additional scenarios with varying assumptions around ZEV and conventional technologies and fuels (see next section).

**Table 1:** Key assumptions used in TCD calculations, *Central* scenario

Parameter	2025	2030	2035	2050
Battery Cost ($/kWh)	217	139	80	50
Battery Energy Density (Wh/kg)	196	223	250	380
Fuel Cell Cost ($/kW)	175	129	80	60
Fuel Cell Specific Power (W/kg)	973	1009	1040	1080
Depot Charging Cost ($/kWh)^a^	0.21	0.18	0.17	0.16
Corridor Charging Cost ($/kWh)^a^	0.36	0.28	0.26	0.24
Hydrogen Cost ($/kg)^a^	9.00	6.00	4.00	4.00
Diesel Price ($/gallon)	3.99	3.65	3.74	3.92
Annual VMT	Varies with vehicle class and market application; see Table S1.1 of the supplemental information			
Time Horizon used in TCD	3 years (Class 3), 4 years (Classes 4–6) or 5 years (Classes 7–8)			
Discount Rate (%)	7			
Opportunity Cost of En-Route Charging Time ($/hour)	75			
EV Recharging Infrastructure	Progressively available with EV adoption; no infrastructure limitations on adoption			
EV Corridor Charging Speed	350 kW (Class 3) or 500 kW (Classes 4–8)			
Hydrogen Refueling Infrastructure	Progressively available with FCEV adoption; no infrastructure limitations on adoption			
Policy	No vehicle purchase subsidies or other supportive policies are assumed			

Vehicle purchase cost and fuel economy specifications are based on simulations conducted by Argonne National Laboratory,^31^ consistent with the component characteristics listed in Table 1. Diesel fuel prices are from the Annual Energy Outlook (AEO), 2023 edition,^2^ while depot and corridor charging costs are estimated inclusive of station cost and utilization.^32^ Hydrogen costs are based on DOE targets and implicitly include investments and incentives.^33^ These and other assumptions are described in more detail in the STAR Methods and supplemental information sections.

a Inclusive of fuel cost and recharging/refueling infrastructure.

To evaluate the competitiveness of different technology options, we compute TCD, considering vehicle purchase costs, fuel costs (inclusive of the levelized cost of infrastructure for EVs and FCEVs), maintenance costs, and the monetized cost of charging time (for EVs) over a three to five-year time horizon. Four powertrains are considered: conventional diesel ICEVs, parallel hybrid electric-diesel vehicles (HEVs), EVs (including three ranges: 150, 300, and 500 miles), and FCEVs. We do not consider plug-in hybrid electric vehicles (PHEVs) in this analysis. We consider vehicle classes 3–8 (10,000 pounds and greater) and market segments encompassing the full scope of US MHDV operations except buses.

**Figure 2:**
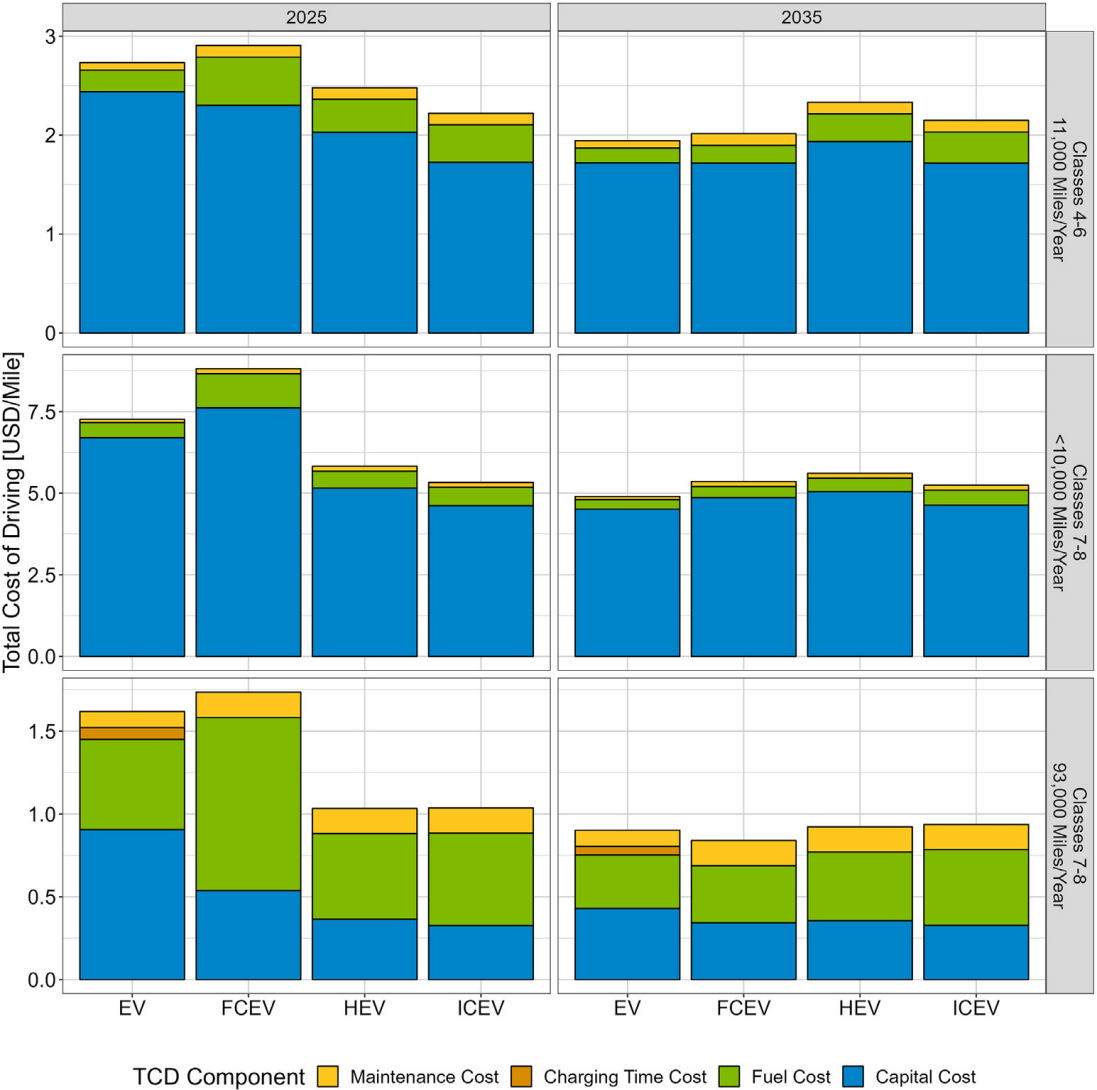
Total Cost of Driving, *Central* Scenario, for Selected Medium and Heavy-Duty Market Segments

Figure 2 compares TCD for various vehicle technologies and selected market segments under central assumptions. For short-haul segments (the majority of light-medium and medium [Classes 3–6] vehicles but a minor share of energy and emissions), capital costs are the largest component of TCD, while fuel costs are the largest component in heavy-duty (Classes 7–8) long-haul segments. EVs have the highest efficiency and lowest maintenance costs, leading to cost savings that increase for high-VMT applications (e.g., long-haul), but tradeoffs can occur due to limited range and the opportunity cost of en-route charging time (here monetized at $75/h; see the supplemental information, Section S3 for a detailed discussion of how opportunity cost is estimated). Figure S5.1 shows TCD for these vehicle technologies for all simulation years.

**Figure 3:**
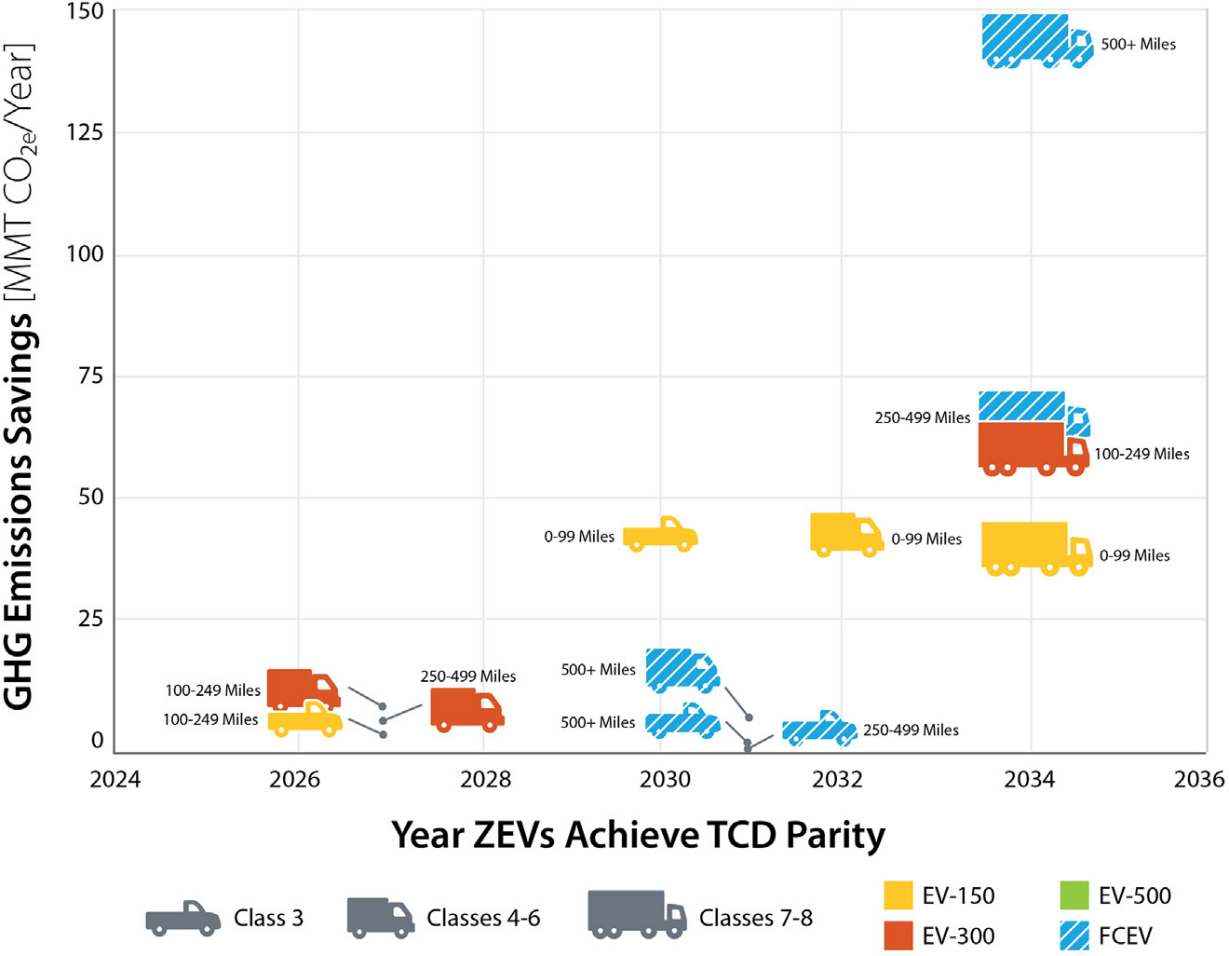
TCD Parity and GHG Emissions Savings Potential by MHDV Class and Market Segment, *Central* Scenario EV labels (EV-150, EV-300, and EV-500) refer to EV range in miles. Mileage labels (0–99 Miles, 100–249 Miles, etc) indicate the primary shipment distance of the market segment. GHG emissions savings potential is estimated from tailpipe CO_2_e emissions in 2019 by vehicle class and shipment distance, and refers to emissions savings if the whole segment were to become zero-emission. TCD parity is computed considering vehicle purchase cost as well as fueling and maintenance costs over the first three to five years of operations. Colors refer to the zero-emission technology that reaches TCD parity with conventional technologies first. Other zero-emission technologies may subsequently reach TCD parity and also become cost competitive, but are not shown. For example, heavy-duty EV-500s in long-haul market segments (500+ mile shipment distance) are not shown but achieve TCD parity between 2035 (for 500–1000 mile shipment distances) and 2045 (for 1000+ mile shipment distances).

Figure 3 plots the time horizon in which ZEVs become competitive on a TCD basis with diesel vehicles (including both ICEVs and HEVs). The interactions between capital cost, operational costs and VMT generally determine when a ZEV technology achieves parity. By 2032 (and in many cases before 2030), ZEVs achieve TCD parity with diesel vehicles in light-medium (Class 3) and medium-duty (Classes 4–6) truck classes, driven by declines in battery costs. Shorter-range EVs with 150–300 miles of electric range reach parity in short-haul and regional market segments, which have lower daily VMT and a reduced need for EVs with larger, costlier batteries. Heavy trucks (Classes 7–8) and trucks that drive longer distances (500+ mile shipment distances) achieve parity after 2030. We find that FCEVs achieve parity earlier than EVs in these market segments, as they are not penalized by the opportunity cost of en-route refueling time. For EVs, time spent on en-route recharging depends on daily driving distance, charging speed and vehicle range. Long-haul Class 7–8 EVs with a 500-mile range and a recharging speed of 500 kW are estimated to spend between ten minutes and two hours refueling per shift (varying with daily mileage and battery size). The opportunity cost of this time is monetized at $75/h (based on Hunter et al.^15^). For all vehicle applications, we find that at least one ZEV technology achieves parity with diesel before 2035. The GHG emissions mitigation potential of ZEV technologies, defined as 2019 tailpipe GHG emissions, is greatest for heavy trucks used in long-haul applications. Heavy trucks in general have the greatest opportunity for emissions mitigation, as they have lower fuel economies and tend to have greater VMT than light-medium and medium trucks. We find that ZEVs achieve parity in these segments by 2034 in the *Central* scenario. In the near-term (by 2030), the greatest mitigation opportunity comes from adopting EVs for Class 3 trucks operating at short distances.

**Figure 4:**
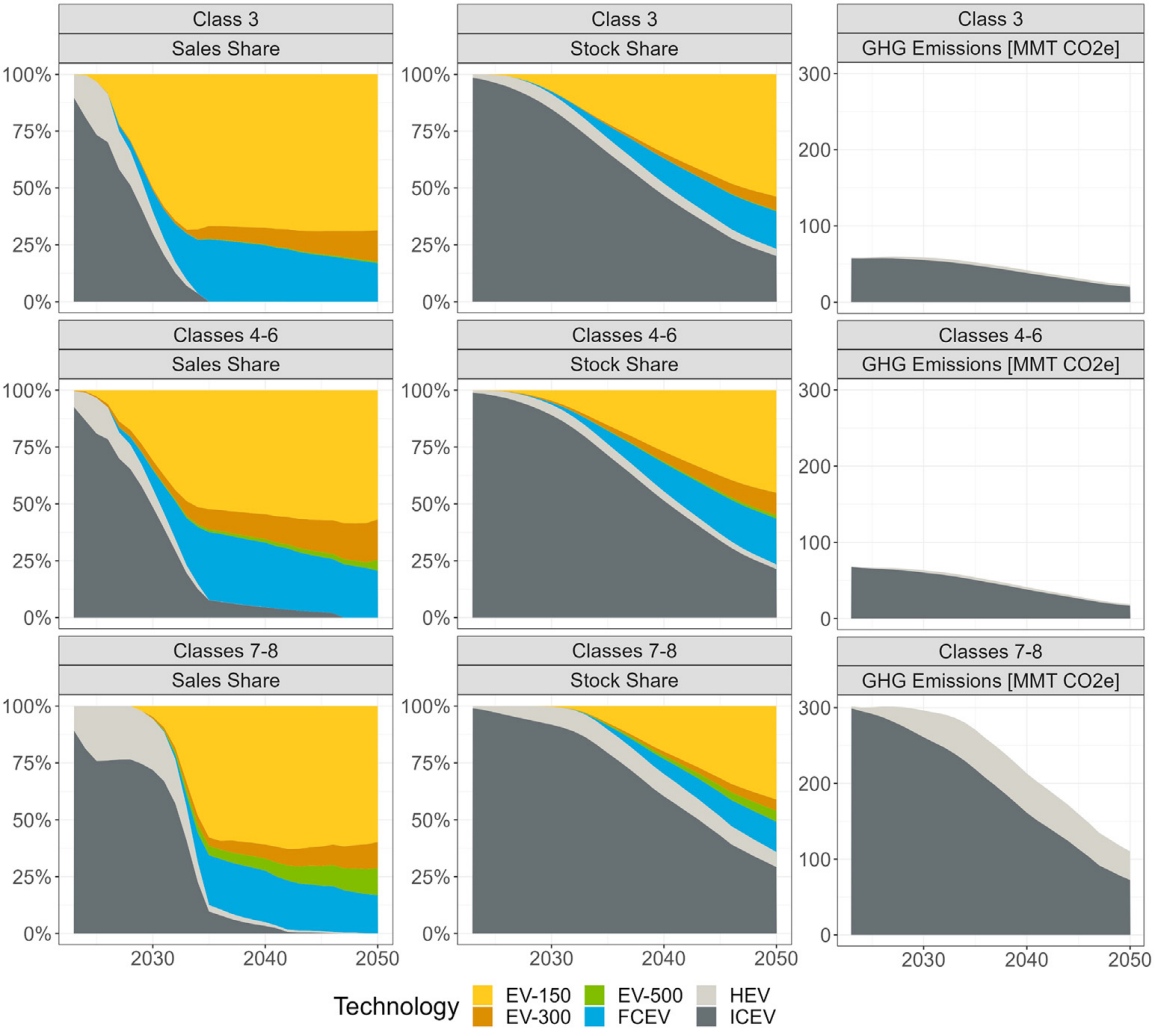
Sales Share, Stock Share, and GHG Emissions by Vehicle Class and Technology in the *Central* Scenario Sales of EVs with 100–300 miles of range grow substantially before 2030 for Classes 3–6, while FCEV sales grow between 2030 and 2050. The greatest emissions reductions come from Classes 7–8 after 2030 as greater numbers of ZEVs are adopted.

Figure 4 shows projected MHDV sales, stock, and emissions over time for the *Central* scenario, computed using the TEMPO model. Vehicle sales are estimated using a logit formulation based on TCD (further described in the STAR Methods section). An integrated vehicle stock model ages existing stock and estimates demand for new vehicles (described in in further detail in the STAR Methods section and Section S4 of the supplemental information). Energy consumption and GHG emissions are estimated based on vehicle stock and VMT in each market segment. Under central assumptions, we estimate that ZEV sales reach 38% in 2030, with the majority concentrated in light-medium and medium vehicle classes and short-range EVs. During this time, total VMT is projected to grow by 10% relative to 2019 levels, based on projections from the Annual Energy Outlook (AEO).^2^^,^^16^^,^^34^ Due to growth in VMT and slow stock turnover, ZEV stock is only 5% in 2030, and emissions decline slightly relative to 2019, by roughly 3%. By 2040, ZEV sales shares reach 97%, resulting in a 41% ZEV stock share and 31% emissions reductions relative to 2019. Sales of FCEVs are projected to increase in the 2030s and 2040s, especially in heavier and longer-distance market segments, accounting for 25% of vehicle sales and 10% of vehicle stock in 2040. By 2045, ZEV sales reach 99%. Stock in 2050 is 73% ZEV (56% EV, 17% FCEV), and emissions reductions are 65% in 2050 relative to 2019, despite 33% growth in projected VMT.

### Impact of IRA vehicle purchase tax credits

We next evaluate the impact of the Commercial Clean Vehicle Credit enacted under the 2022 IRA. The Commercial Clean Vehicle Credit (Provision 45W) is a tax credit equal to the lesser of 15% of the vehicle purchase price for plug-in hybrid electric vehicles (not considered in this study), 30% of the vehicle purchase price for EVs and FCEVs, or the incremental cost of the vehicle compared to an equivalent ICEV, up to $40,000 for Classes 4–8 and $7,500 for Class 3.^35^ We incorporate this tax credit into our TCD analysis but do not explicitly model other aspects of the IRA that affect fuel and component prices, including subsidies for renewable electricity and hydrogen production (Provision 45/45Y and 45V), subsidies for refueling infrastructure (Provision 30C) and subsidies for batteries manufactured in the United States (Provision 45X). However, our central assumptions assume improvements in ZEV technologies and in hydrogen cost aligned with DOE scenarios that implicitly require investments and incentives to achieve.

**Figure 5:**
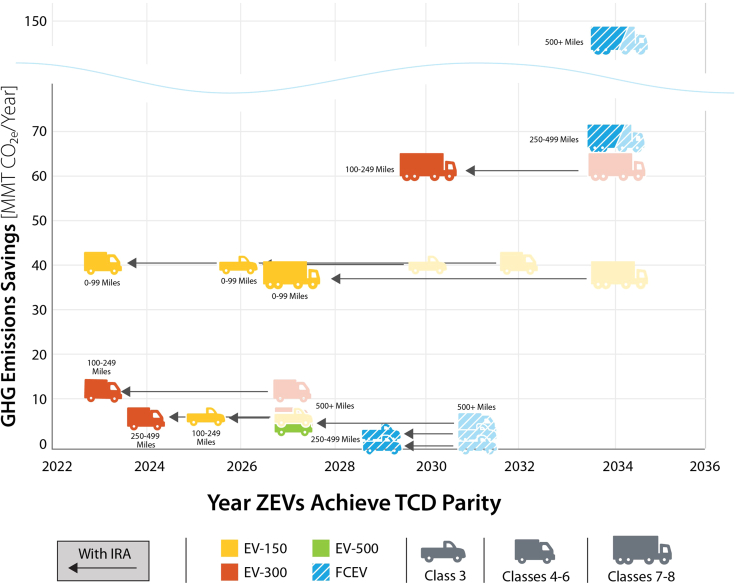
Impact of the Inflation Reduction Act Commercial Clean Vehicle Credit (Provision 45W) on ZEV TCD Parity Year by MHDV Class and Market Segment The Commercial Clean Vehicle Credit greatly accelerates the time at which ZEVs reach TCD parity, enabling market uptake in the near term. EV labels (EV-150, EV-300, and EV-500) refer to EV range in miles. Mileage labels (0–99 Miles, 100–249 Miles, etc) indicate the primary shipment distance of the market segment. GHG emissions savings potential is estimated from tailpipe CO_2_e emissions in 2019 by vehicle class and shipment distance, and refers to potential emissions savings if the whole segment were to become zero-emission. TCD parity is computed considering vehicle purchase cost as well as fueling and maintenance cost over the first 3–5 years of operations. Colors refer to the zero-emission technology that reaches cost parity with conventional technologies first. Other zero-emission technologies may subsequently reach TCD cost parity and also become cost competitive, but are not shown.

With vehicle purchase credits, ZEVs, particularly EVs, achieve TCD parity with diesel vehicles on substantially earlier time frames (Figure 5). For light-medium vehicles, a majority of vehicles achieve TCD parity by 2026 (1–4 years earlier than under central assumptions). Assuming no lag in adoption occurs as the market adjusts, this results in an additional 0.7 million light-medium vehicles sold, 48 billion VMT driven on ZEVs and tailpipe emissions savings of 33 MMT CO_2_e between 2023 and 2032. For medium vehicles, a majority of vehicles achieve TCD parity by 2023 or 2024 (3–9 years earlier than under central assumptions), resulting in an additional 1.1 million vehicles sold, 81 billion VMT driven on ZEVs, and tailpipe emissions savings of 73 MMT CO_2_e. For heavy vehicles, short-haul market segments achieve parity between 2027 and 2030 (versus 2034 without incentives). Heavy regional and long-haul market segments continue to achieve parity in 2034, with FCEVs remaining the most cost competitive on a TCD basis. The lack of change in time to reach TCD parity is because FCEVs’ primary barrier to TCD competitiveness is their high fuel cost, which remains unchanged from the *Central* scenario. For EVs the reductions in purchase cost are not high enough to reach TCD parity between 2023 and 2032. However, EVs get closer to TCD parity and achieve a higher market share with IRA vehicle purchase credits. This results in a total of 0.9 million additional vehicles sold, 23 billion additional miles driven on ZEVs, and savings of 29 MMT CO_2_e across heavy long and short-haul market segments. After 2032, when we assume the tax credits expire, ZEV sales follow the same trajectory as under central assumptions, as vehicle purchase prices return to the central trajectory. This scenario does not consider additional cost reductions caused by manufacturing scale up or learning that could be induced by IRA tax credits. With IRA tax credits, ZEV stock increases from 5% in the central scenario to 20% in 2030, resulting in emissions reductions of 8% in 2030 relative to 2019 (versus a 3% reduction in the central scenario). In 2040, ZEV stock increases from 41% in the central scenario to 55% with IRA tax credits, resulting in emissions reductions of 39% relative to 2019 (versus 31% in the central scenario). Finally, in 2050, ZEV stock increases from 73% in the central case to 81% with the IRA, while emissions are reduced by 70% relative to 2019 (versus 65% in the central scenario). Cumulatively, this results in 6% lower MHDV GHG emissions from 2019 to 2050 compared to the central scenario.

### Alternative scenarios: Fuels and technologies

Our results are highly sensitive to assumptions surrounding future technology attributes and fuel costs, which are highly uncertain. For short-haul applications of all classes, upfront capital cost is the greatest driver of TCD, while for long-haul applications, fuel costs account for greater shares of TCD (see Figure 2). To better understand the impact of fuel costs and future technology evolution on ZEV adoption, we evaluate a set of ten scenarios exploring variations on our central assumptions, including more conservative ZEV technology progress, more advanced technology progress for diesel technologies, and varied fuel cost assumptions (see Table 2).

**Table 2:** Fuel and technology scenarios

Scenario	Description
Cons. ZEV Tech Progress	ZEV purchase costs and fuel economy follow the conservative technology progress trajectory from Islam et al.^31^
Adv. ICEV & HEV Tech Progress	ICEV and HEV purchase costs and fuel economy follow the advanced technology progress trajectory from Islam et al.^31^
No EV-150	EVs with a 150-mile range are considered infeasible by vehicle operators for operations in all classes and market segments
Low Diesel	Diesel prices decline to $2.3/gallon by 2027, based on the historical minimum from 2008 to 2019^36^
High Diesel	Diesel prices reach $6/gallon by 2040, following the AEO *High Oil Price* trajectory^2^
Cons. Electricity	Corridor charging costs reach $0.37/kWh by $2035 and $0.35/kWh by 2050, while depot charging costs reach $0.21/kWh by $2035 and $0.2/kWh by 2050, reflecting higher infrastructure cost and lower utilization
Adv. Electricity	Corridor charging costs reach $0.19/kWh by $2035 and $0.16/kWh by 2050, while depot charging costs reach $0.15/kWh by $2035 and $0.13/kWh by 2050, reflecting lower infrastructure cost and higher utilization
Cons. Hydrogen	Hydrogen costs reach $7/kg by 2030 and do not improve further in subsequent years
Adv. Hydrogen	Hydrogen costs reach $4/kg by $2035 and $3/kg by 2040
Cons. Hydrogen & Electricity	Combines the assumptions of the *Cons. Electricity* and *Cons. Hydrogen* sensitivities

**Figure 6:**
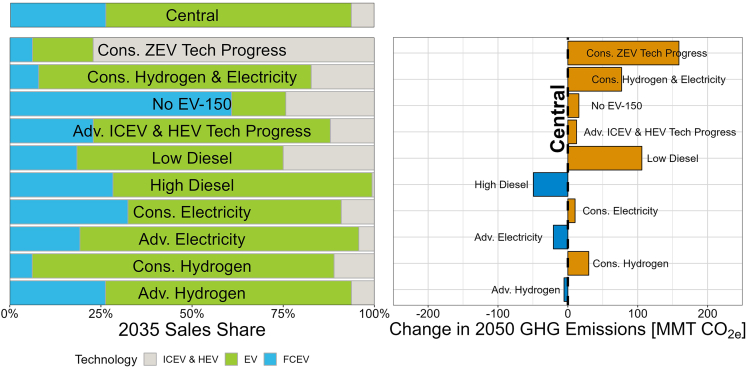
Sensitivity of 2035 MHDV Sales and 2050 GHG Emissions to Altered Assumptions, Compared to the *Central* Scenario Note that sales shares and emissions are not necessarily proportional to one another due to differences in VMT and fuel economy across market segments and vehicle classes.

Figure 6 plots the GHG emissions implications of these alternative scenarios relative to our central assumptions. We find that that the *Cons. ZEV Tech Progress* scenario, which assumes a slower rate of ZEV capital cost reductions and fuel economy improvements, results in the greatest 2050 emissions increases (159 MMT CO_2_e increase over central assumptions, resulting in only a 28% reduction in GHG emissions relative to 2019) and the lowest ZEV sales (23% ZEV sales in 2035 and 70% in 2050, compared to 94% and 100% under central assumptions). If instead ICEV technology improves more rapidly, resulting in lower capital costs and improved fuel economy (the *Adv. ICEV & HEV Tech Progress* scenario), there is a smaller impact on emissions and ZEV adoption, with an additional 12 MMT CO_2_e emissions, 87% ZEV sales in 2035 and 99% ZEV sales in 2050. Lower diesel prices based on historical minimum prices (the *Low Diesel* scenario) have the second most substantial impact on emissions, resulting in a 106 MMT increase in GHG emissions (a 40% reduction relative to 2019) and a 2035 ZEV sales share of 75% (95% by 2050). A combination of conservative hydrogen and electricity costs (the *Cons. Hydrogen & Electricity* scenario), which could occur due to factors such as higher fuel production and refueling station costs and lower station utilization, have a similar impact, resulting in a 77 MMT increase in 2050 GHG emissions relative to central assumptions. Other sensitivities have less impact on total GHG emissions and instead may alter the tradeoffs between EVs and FCEVs. Conservative hydrogen or electricity costs (the *Cons. Hydrogen* and *Cons. Electricity* scenarios) increase 2050 GHG emissions by 10–30 MMT, as sales of either FCEV or EV technologies are often replaced by the other technology. Optimistic hydrogen or electricity assumptions have a similar magnitude in the opposite direction; the *Adv. Electricity* scenario reduces 2050 emissions by 21 MMT over central assumptions and results in higher 2050 EV stock (62% EV and 12% FCEV), while the *Adv. Hydrogen* scenario reduces 2050 emissions by 5 MMT and results in higher FCEV stock (51% EV and 22% FCEV). Among more optimistic sensitivities (more favorable electricity, hydrogen, or diesel price assumptions), we find that higher diesel prices (averaging $6/gallon rather than $4/gallon from 2023 to 2050) result in the greatest emissions reductions relative to central assumptions (an additional 49 MMT in 2050, or emissions reductions of 76% relative to 2019).

We additionally consider a scenario in which EVs with 150 miles of range (EV-150s) are not considered viable by fleet operators due to their limited range (the *No EV-150* scenario). This scenario is meant to explore implications of excluding shorter range EVs as technology options due to possible concerns such as range anxiety or range requirements that prevent EV-150s from being viable. Excluding EV-150s from the set of possible M/HDV technology options substantially reduces ZEV sales in the near-term (13% ZEV sales in 2030, compared to 38% in the central scenario) and alters the balance between EVs and FCEVs. For shorter-distance applications more FCEVs are purchased rather than longer-range EVs because of the high upfront costs of longer-range EVs, which are less competitive in low-mileage market segments. Long-run emissions are not substantially impacted because of substitution between short-range EVs and FCEVs. ZEV sales reach 88% by 2040 and 99% by 2050, while 2050 emissions are reduced by 61% relative to 2019. FCEV stock grows to 43% by 2050, while EV stock is 21%, with the remainder being ICEVs and HEVs.

**Figure 7:**
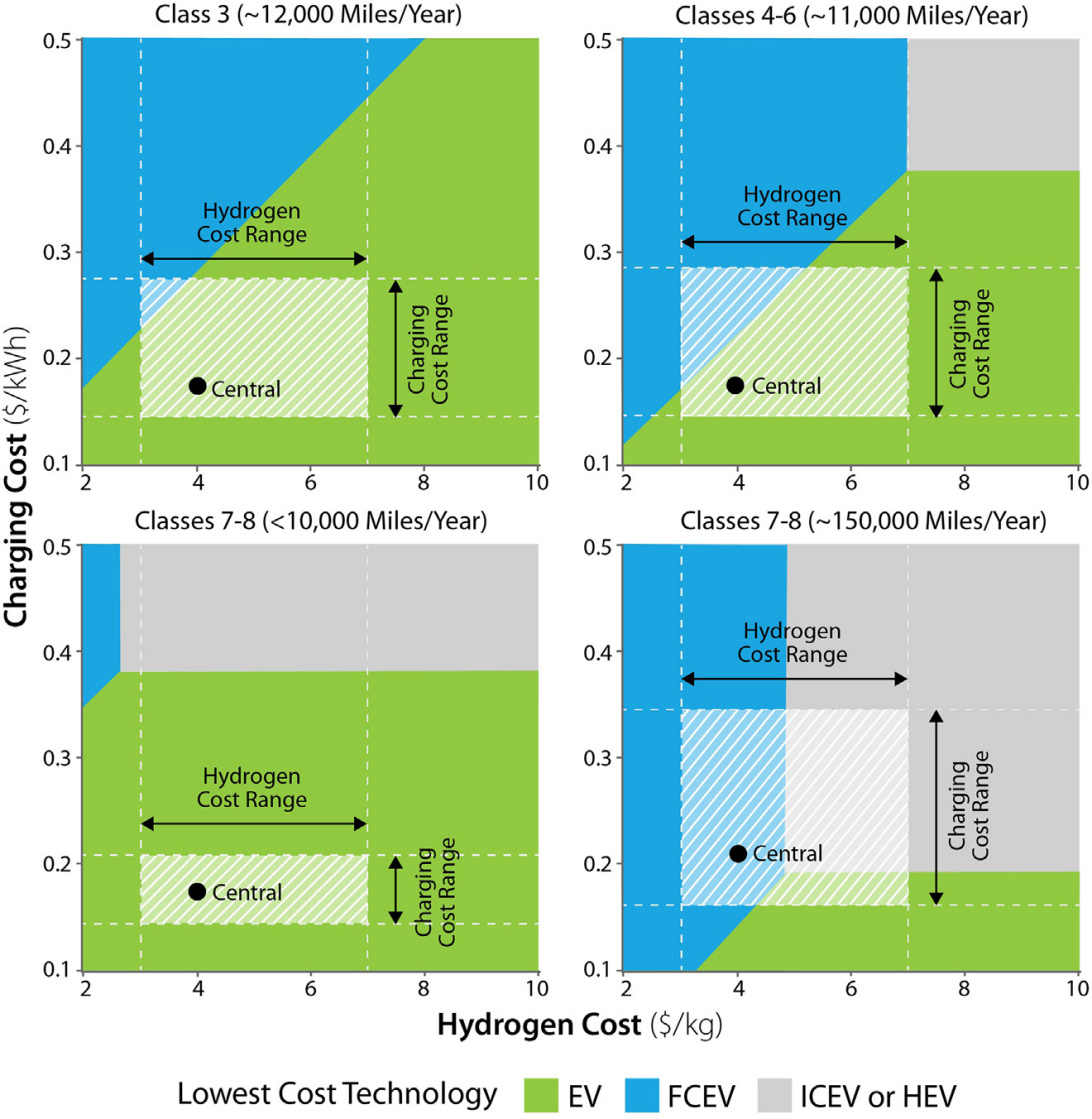
Fuel Cost Sensitivities, 2035, *Central* Technology Assumptions and Selected Market Segments Results assume $4/gallon diesel and 0.5 MW charging. In selected short-haul market segments, all EV classes are competitive on a TCD basis with diesel vehicles at charging costs below $0.35 to $0.4/kWh. FCEVs are competitive on a TCD basis in light-medium and medium segments at hydrogen costs below $7/kg, and below $5/kg in heavy long-haul segments. This scenario does not include the impact of IRA vehicle purchase tax credits.

Figure 7 illustrates the sensitivity of our central findings to altered fuel cost assumptions in 2035 for selected market segments. These findings do not include the impact of IRA vehicle purchase tax credits. Both EVs and FCEVs can become competitive on a TCD basis in multiple market segments, offering alternative and in some cases complementary options for decarbonization. In short-haul light-medium and medium market segments, EVs with 150–300 miles of range are competitive on a TCD basis with diesel vehicles for all vehicle classes at charging costs below $0.35 to $0.4/kWh (averaged over both corridor and depot charging). FCEVs are competitive on a TCD basis below $6-$7/kg. For heavy vehicles, there is more heterogeneity across market segments. In short-distance applications (below 10,000 miles per year), EV-150s are competitive at average charging costs below $0.38/kWh, while for other short-haul and regional applications (driving 68,000 to 93,000 miles per year) this cost varies from $0.18 to $0.25/kWh. In long-haul market segments driving more than 100,000 miles per year, longer-range (500 mile) EVs become competitive with diesel vehicles at average charging costs below $0.17 to $0.18/kWh for a charging speed of 0.5 MW as a result of tradeoffs between capital cost, opportunity costs from en-route charging time, and fuel cost savings. When a 1 MW charging speed is assumed rather than 0.5 MW (reducing opportunity costs from en-route charging), long-range EVs are competitive in long-haul market segments at average charging costs below $0.22/kWh. We note that this segment may have diverse operational and refueling requirements (for example, team and multi-shift driving versus single-shift), which may result in a diversity of solutions and price points; the results presented here are average estimates. FCEVs require hydrogen costs (dispensed to the vehicle) of $5/kg to become cost competitive in most heavy-duty market segments on a TCD basis. Supplemental figures show cost competitiveness on a TCD basis for all heavy market segments at charging speeds of 0.5 and 1 MW in 2035 (Figures S5.2 and S5.3). These findings are sensitive to technology costs and diesel prices; in 2050, when ZEV technology progress is assumed to have further advanced, heavy long-haul vehicles are competitive at charging costs of $0.26/kWh or lower ($0.29/kWh or lower for 1 MW charging), and FCEVs are competitive at hydrogen costs of $6/kg (Figure S5.4). The market share of EVs and FCEVs is highly sensitive to fuel costs for long-haul segments; increasing hydrogen costs or decreasing charging costs change the trade-offs between the two technologies.

## Discussion and conclusions

We evaluate the economic competitiveness of EVs and FCEVs in the entire US MHDV sector on a TCD basis and the possible implications for vehicle sales, stock evolution, energy use, and GHG emissions. Using government and industry-vetted technology cost and fuel price projections aligned with achieving US DOE high technology progress scenarios, our findings show that ZEV technologies, including both EVs and FCEVs, can become competitive on a TCD basis with conventional and hybrid diesel technologies prior to 2035 across multiple vehicle classes and market segments. For light-medium and medium vehicle classes (Classes 3–6), EV TCD competitiveness is achieved by 2032 for all vehicles and applications for the *Central* scenario and across many sensitivities. For heavy vehicle classes, EVs achieve TCD competitiveness in all short-haul and some long-haul market segments by 2035 under *Central* scenario assumptions. In 2035, EVs are cost-competitive on a TCD basis in all light-medium, and medium short-haul market segments under central assumptions when charging costs are below $0.35 to $0.4/kWh and are competitive in all heavy segments at charging costs of $0.17/kWh or less (with some segments competitive at costs of up to $0.38/kWh). Because of the penalty imposed for charging times, faster charging speeds (1 MW instead of 0.5 MW) increase EV TCD competitiveness in regional and long-haul segments, allowing them to compete with diesel vehicles at charging costs of $0.22/kWh or less in 2035. FCEVs could become cost-competitive on a TCD basis prior to 2035 in heavy long-haul market segments at hydrogen costs below $5/kg and can become competitive in some light-medium and medium short-haul applications during this time frame if hydrogen costs are below $7/kg. Technology transitions result in 38% ZEV sales by 2030 and 99+% by 2046 in our *Central* scenario. If TCD drives vehicle adoption (i.e., the cheapest technology solution gets adopted in each vehicle class and segment), demand for ZEVs could rapidly increase once TCD parity is reached. This rapid scale up in ZEV sales, going from almost no market share to full dominance in about a decade, would require major scale-ups for charging and refueling infrastructure, manufacturing capacity, workforce, and the economy that were not considered here, but are the subject of separate efforts. For example, Knehr et al. anticipates that sufficient supply for US-manufactured automotive lithium-ion batteries will be available to meet demand for light, medium, and heavy-duty vehicles between the present and 2035, based on current manufacturer announcements.^37^ Atlas Public Policy estimates that current, planned and potential production capacity for ZEVs could produce up to 295,000 heavy-duty ZEVs per year, which would account for 59 percent of annual US heavy-duty vehicle sales, though no time frame is given.^38^ In addition, the US DOE estimates that there is existing or planned capacity to produce over one million chargers per year in the United States, including 60,000 fast chargers.^39^ These studies indicate that the industry is addressing the multiple manufacturing and supply chain transformations needed to enable a rapid scale-up in ZEV deployment.

By 2050, a rapid transition to ZEVs results in substantial GHG emissions reductions – 65% relative to 2019 levels without policy and 70% with IRA vehicle purchase tax credits considered. Policies to accelerate vehicle turnover or to reduce GHG emissions from conventional technologies (such as sustainable liquid fuels) could further reduce emissions. Consistent with previous research such as Hunter et al.^15^ and Burke et al.,^24^ we find that EVs are most competitive on a TCD basis in lighter and shorter-range market segments, while FCEVs are competitive in long-haul segments, as longer recharging times and higher corridor charging costs for EVs impose substantial costs. In short-haul segments, EVs are the least cost technology at charging costs of up to $0.5/kWh (higher costs were not explored in this analysis). In regional and long-haul segments, FCEVs are cost competitive on a TCD basis with diesel when hydrogen costs are below $6/kg; EVs are cost competitive on a TCD basis when charging costs are below $0.26/kWh (or $0.28/kWh with 1 MW charging). The infeasibility of EVs with ranges of 150 miles would substantially reduce ZEV sales in the near-term and alters the balance between EVs and FCEVs because of the high upfront costs of longer-range EVs, which are less competitive in low-mileage market segments. Overall, the tradeoffs between EVs and FCEVs largely depend on uncertain technology costs, especially for batteries, as well as hydrogen costs and the cost of charging EVs, which is expected to vary significantly for different applications, over time, and across regions.^40^^,^^41^ This uncertainty demonstrates the need for further pilot demonstrations and data collection for both EV and FCEV technologies in various MHDV applications. Deployment of EV charging and hydrogen refueling infrastructure will be required to support EV and FCEV adoption, with regional deployment of infrastructure at depots and fixed routes required in the near-term (prior to 2030) and progressive build-out of corridor networks on longer time horizons. Charging and refueling costs will be substantially influenced by economies of scale and station utilization rates; efficient siting, favorable electricity rate designs, and high-volume production of fuels (for hydrogen) are necessary to achieve competitive costs.

Policy can further accelerate technology transitions and reduce emissions. The Commercial Clean Vehicle Credit (Provision 45W) of the Inflation Reduction Act of 2022 can reduce the time needed for ZEVs to achieve TCD competitiveness by up to nine years, effectively ensuring a strong demand for clean technologies (with adoption likely limited by supply and infrastructure limitations). While not modeled separately, the hydrogen production tax credit can also significantly reduce hydrogen costs, making FCEV technologies also competitive for certain applications, highlighting the rapid changes likely to happen. The emissions implications of accelerating this transition are substantial; emissions reductions in 2050 increase from 65% to 70% relative to 2019, while cumulative emissions fall by 6%. These vehicle tax credits primarily support short-haul EV deployment, as they are closer to parity with ICEVs. Long-haul EV deployment may be supported by expanded infrastructure, fast recharging or refueling times, and reduced fuel costs in addition to vehicle purchase price reductions modeled in this study. Enabling low electricity and hydrogen costs is a key determinant of the TCD competitiveness of long-haul ZEVs.

Substantial uncertainties are inherent in our analysis, including future technology evolution and fuel costs. Our central findings are contingent on a high level of technology progress for ZEVs, with battery pack costs reaching $50/kWh and fuel cell costs reaching $60/kW by 2050 and hydrogen reaching $4/kg by 2035, inclusive of production, transport, and dispensing. More conservative ZEV technology progress substantially delays the TCD competitiveness of zero-emission MHDVs, resulting in 2050 emissions reductions of only 28% relative to 2019, rather than 70% under central assumptions. More pessimistic assumptions for hydrogen and electricity costs also substantially impact results, but cost changes in only one fuel or the other are less impactful. The reduced impact of relative fuel costs is because EVs and FCEVs compete with one another for market share; a change in operating costs for one technology results in the other taking a greater share of sales. Finally, high diesel prices have a substantially positive impact on sales of both ZEV technologies.

This study offers a unique sector-wide perspective on the TCD competitiveness and decarbonization potential of commercial MHDVs in the United States. Distinct from previous research, we consider the full US commercial vehicle market, covering all truck classes for freight movement and other uses (e.g., vocational vehicles). We find that with continued progress in zero-emission technology and fuels, large-scale transformations of the MHDV sector are feasible based on economics and can be further accelerated through tax credits, resulting in substantial decarbonization by mid-century. We show that from an economic perspective, ZEV markets are on the verge of becoming rapidly mature across a wide range of MHDV applications, especially when IRA incentives are considered. The expectation that demand for medium and heavy-duty ZEVs could substantially increase over the next few years calls for forward-looking investments to enable adoption, including infrastructure and manufacturing scale-up, grid preparedness and multi-stakeholder engagement to enable the realization of major cost savings and emissions reductions.

### Limitations of the study

This analysis estimates potential ZEV sales based on TCD but does not consider other factors that may influence adoption. Our scenarios suggest that the demand for new vehicles could increase rapidly, particularly between 2030 and 2035, when ZEV sales shares increase from 38% to 94%. In practice, this expansion could be constrained by limitations in vehicle supply, charging and refueling infrastructure, workforce constraints, or other supply related factors.^42^^,^^43^ A second but related consideration is the market for used vehicles. We assume that vehicles remain in their designated market segment for their entire lifetime; in practice, used vehicles may transfer between market segments over their lifetime (such as heavy-duty vehicles shifting from long to short-haul applications as they age). This assumption may have a positive or negative impact on the rate of adoption, depending on the rate of adoption in the initial market segment in which vehicles are sold. Finally, we do not consider future logistical changes in the freight sector, such as shifts between rail, marine, and on-road transport modes or changes in truck travel behavior and trip lengths. Areas for future research include analysis of potential market transformations such as mode shifting and logistical changes, detailed analysis of infrastructure and charging needs to fully support ZEV adoption, further analysis of vocational vehicle operational profiles, and analysis of the co-evolution of on-road and supply-side emissions as ZEV adoption increases, including in the electricity and hydrogen production sectors.

## STAR★Methods

### Key resources table

**Table:** 

REAGENT or RESOURCE	SOURCE	IDENTIFIER
**Deposited data**		

Vehicle Inventory and Use Survey, 2004 edition	U.S. Census Bureau^18^	https://www.census.gov/library/publications/2002/econ/census/vehicle-inventory-and-use-survey.html
Freight Analysis Framework, Version 5	Federal Highway Administration^17^	https://faf.ornl.gov/faf5/Default.aspx
Annual Energy Outlook, 2019, 2021 & 2023 editions	Energy Information Administration^2^^,^^16^^,^^37^	https://www.eia.gov/outlooks/aeo/data/browser/
M/HDV vehicle cost and fuel economy trajectories, Autonomie model	Islam et al.^31^	https://anl.app.box.com/s/an4nx0v2xpudxtpsnkhd5peimzu4j1hk/folder/177858439896
Maintenance costs	Hunter et al.^15^	https://www.nrel.gov/docs/fy21osti/71796.pdf
Historical diesel prices	Energy Information Administration^39^	https://www.eia.gov/dnav/pet/hist/LeafHandler.ashx?n=PET&s=EMD_EPD2DXL0_PTE_NUS_DPG&f=A
Hydrogen costs	NREL, based on U.S. DOE targets^35^	https://data.nrel.gov/submissions/227; https://doi.org/10.7799/2283767
Depot and corridor charging costs	NREL, forthcoming.^34^	https://data.nrel.gov/submissions/227; https://doi.org/10.7799/2283767

**Software and algorithms**		

Transportation Energy & Mobility Pathway Options™ (TEMPO) Model	NREL	https://www.nrel.gov/transportation/tempo-model.html
Julia version 1.2.0	Julia Programming Language	https://julialang.org/
R	The R Foundation	https://www.r-project.org/

### Resource availability

#### Lead contact

Further information and requests for resources should be directed to and will be fulfilled by the lead contact, Catherine Ledna (catherine.ledna@nrel.gov).

#### Materials availability

This study did not generate new datasets.

#### Data and code availability

• Model input data and results for all scenarios have been deposited at https://data.nrel.gov/submissions/227 and are publicly available as of the date of publication. DOIs are listed in the key resources table.
• The results generated in this study leveraged the National Renewable Energy Laboratory’s Transportation Energy & Mobility Pathways™ (TEMPO) model. The model has been peer-reviewed and is documented and validated, in M. Muratori et al., “Exploring the Future Energy-Mobility Nexus: The Transportation Energy & Mobility Pathway Options (TEMPO) Model,” *Transp. Res. Part D Transp. Environ.* 98, 102967 (2021). More details on the code are available from the lead author on reasonable request.
• Any additional information required to reanalyze the data reported in this paper is available from the lead contact upon request.

### Method details

#### Scope and market segmentation

Our scope includes all commercial MHDVs with a GVWR of over 10,000 pounds, with the exception of buses. We divide MHDVs into three vehicle classes (light-medium – Class 3, medium – Classes 4-6, and heavy – Classes 7-8) and up to eight market segments for both freight and non-freight vehicles. To do this, we integrate several data sources, including FAF,^17^ the 2002 VIUS,^18^ and the 2019 AEO.^16^

We first disaggregate initial (2017) vehicle population and VMT by class from the AEO into freight and non-freight categories using freight and non-freight vehicle and VMT fractions estimated from a synthesis of VIUS and IHS-Polk data.^44^ These fractions are listed in Table S2.2. Total freight and non-freight vehicle populations and VMT are estimated by multiplying these fractions by total vehicle stock and VMT by class from the AEO. Freight vehicles and activity are divided into up to eight market segments based on FAF’s shipment distance bins, whose distances represent the movement of goods from origin to destination. These market segments are assumed to differ by daily and annual VMT, which in turn influence vehicle requirements (e.g., range) and operational costs (i.e., fuel and maintenance costs as well as the opportunity cost of dwell times to recharge EVs). Freight movement data from FAF (expressed in ton-miles by shipment distance bin) is combined with vehicle population shares and load factor data (in ton-miles per vehicle) from VIUS to develop estimates of vehicle activity by vehicle class and market segment (average daily and annual VMT). Vehicle population shares by class and market segment are computed from VIUS’ share of vehicles by class and primary operating distance. Table S2.1 describes the mapping between FAF’s shipment distance bin and VIUS’ primary operating distance. Table S1.1 describes average daily and annual VMT by vehicle class and market segment resulting from this synthesis.

Freight vehicle stock by class and market segment is estimated by multiplying the vehicle population share from this FAF-VIUS synthesis by the total freight population estimate by class from the AEO. VMT in each market segment is estimated using load factors from VIUS and ton-miles from FAF. Load factors are adjusted to ensure alignment between total VMT and ton-miles in the AEO and FAF. We validate our approach by comparing the distribution of VMT by vehicle class in TEMPO and VIUS (Figure S2.1). We find that our distributions are largely similar, validating our decision to map VIUS primary operating distance to FAF shipment distance when developing TEMPO’s market segmentation.

Non-freight vehicles are assumed to operate in the 0-99 and 100-249 mile market segments. Non-freight vehicles and VMT are split between these two segments using the same proportions as short-haul freight. Due to a lack of data on non-freight operational constraints, we assume that sales for non-freight vehicles are the same as those of freight vehicles in their respective distance bins. In future years, freight and non-freight stock and activity are assumed to grow at the rate of projected VMT in the 2023 AEO^2^ (Figure S1.6).

#### Framework for estimating total cost of driving

We estimate the total cost of driving (TCD) of competing ZEV and conventional technologies based on bottom-up analysis of vehicle, fuel, and maintenance costs. TCD includes the upfront vehicle purchase cost and future operating costs (considered over a fixed time horizon), and is computed according to the following equations:

(Equation 1) $${TCD}_{c,t,s,y}=\frac{k_{c,t,y}\times crf_{c}}{{VMT}_{c,s}\times load_{c}}+\frac{{\left(\frac{p_{t,y\;}}{fe_{c,t,y}\times eff}\right)+m}_{c,t}{+v}_{c,t,s,y}}{load_{c}}$$

(Equation 2) $$crf_{c}=\frac{\left[d\times {\left(1+d\right)}^{h_{c}}\right]}{\left[{\left(1+d\right)}^{h_{c\;}}-1\right]}$$

TCD (Equation 1) is computed for vehicle class *c,* technology *t,* market segment *s,* and year *y,* with units of U.S. dollars (USD) per ton-mile*.* Upfront capital cost is levelized based on an assumed discount rate *d* and financial horizon *h* (varying with vehicle class), where *k* is the upfront capital cost*,* $crf_{c}$ is the capital recovery factor (Equation 2)*,* ${VMT}_{c,s}$ is the annual vehicle-miles traveled per vehicle, and $load_{c}$ is the vehicle load factor in tons per vehicle. Operating costs are computed from fuel cost $p_{t,y\;}$ (in USD per unit energy)*,* fuel economy $fe_{c,t,y}$ (miles per unit energy), charging efficiency eff (unitless), maintenance cost $m_{c,t}$ (in USD per mile) and the opportunity cost of en-route recharging time $v_{c,t,s,y}$ (in USD per mile). This value is divided by the vehicle’s load factor to convert to USD per ton-mile. We do not consider differences in resale value across powertrains, given the high uncertainty and lack of data. Distinct from estimates of the total cost of ownership, we omit factors such as driver wages and insurance that do not very significantly across powertrain technologies and thus have limited effects on adoption decisions. Table S1.2 includes a detailed list of TCO assumptions and related sources. Section S3 of the supplemental information further documents our methods to estimate the opportunity cost of en-rotue recharging time for EVs.

#### Framework for estimating vehicle sales, stock and emissions

The Transportation Energy & Mobility Pathway Options (TEMPO™) model is used to estimate MHDV sales, stock, energy, and GHG emissions. Muratori et al.^30^ contains additional information on the model and its validation. VMT projections from 2017 to 2050 are taken from the AEO, 2019, 2021 and 2023 editions.^2^^,^^16^^,^^34^ Future freight activity in ton-miles is assumed to grow at the rate of VMT, assuming class-specific vehicle load factors that are held constant over time. Vehicle stock is assumed to increase proportionally to VMT, while total vehicle sales are estimated based on demand and vehicle retirement. Vehicles are assumed to retire following class-specific retirement curves based on trajectories provided by the Energy Information Administration^45^ (Figure S4.1). Total vehicle sales and stock are validated against projections from the AEO (Figures S4.2 and S4.3), while historical energy consumption is compared to 2019 estimates (Table S4.1). Vehicle sales approximately match AEO trajectories by class, with some year-to-year fluctuations. Vehicle stock is slightly lower than AEO for light-medium and heavy vehicle classes, likely due to the assumption of constant VMT and ton-miles per vehicle in TEMPO. Due to a lack of data, we do not model the transfer of used vehicles between market segments, instead assuming that vehicles remain in their original segment for the duration of their lifetime.

Sales shares for each vehicle class and shipment distance are estimated using a logit formulation based on the TCD of competing powertrains, assuming that that economics is the main driver of adoption of different technologies. We assume that if multiple powertrains cost the same on a TCD basis, they will split the market evenly. When a technology achieves a critical TCD competitiveness threshold (roughly 10 to 15% cheaper than other alternatives), we assume that the technology will capture 90% of the market. Logit parameters are calibrated for each market segment to ensure that at least five years of cost savings are achieved before 95% sales of a ZEV powertrain can be reached. This logit formulation allows us to implicitly capture some market heterogeneities – i.e., early adopters within a market segment, who may adopt emerging powertrains before they achieve TCD parity with conventional vehicles, and late adopters who may be more hesitant to adopt new powertrains even if they could produce cost savings. Sales shares are computed based on the following formulation:

(Equation 3) $$w_{c,t,s,y}=e^{\left(-\beta_{c,s}\times u_{c,t,s,y}\right)}$$

(Equation 4) $${sales}_{c,t,s,y}=\frac{w_{c,t,s,y}}{\sum_{t=1}^{T}w_{c,t,s,y}}$$

Where *w* is the weight of technology *t* for vehicle class *c*, market segment *s,* and year *y,* β is the logit cost coefficient for that class and market segment, *u* is total cost of driving (computed as in Equation 1 and normalized relative to its competitors), and *sales* is the sales share.

#### Scenario design and input assumptions

We develop a set of scenarios including the *Central* scenario and ten alternative futures for technology and fuel progress, based on bottom-up trajectories developed by the US DOE and vetted with industry. Central technology assumptions (upfront capital cost and fuel economy) are based on powertrain simulations developed by Argonne National Laboratory using the Autonomie model.^31^ These assumptions correspond to battery pack price targets of $80/kWh by 2035 and $50/kWh by 2050 and fuel cell targets of $80/kW by 2035 and $60/kW by 2050 (Figure S1.1). Over the same time period, fuel economy for diesel powertrains (ICEV and HEV) is assumed to improve by 32 to 37 percent, varying with vehicle class. Because Autonomie simulations span a more detailed set of vehicle classes and vocations than are represented in TEMPO, technology attributes are aggregated using the representative vehicles specified in Table S1.4. TEMPO vehicle cost and fuel economy inputs are plotted in Figures S1.2 and S1.3. Our central assumptions use conservative technology progress trajectories for diesel powertrains and advanced technology progress trajectories for ZEV powertrains, reflecting a future in which resources are allocated toward research and development of ZEVs. Sensitivities explore permutations on these assumptions, including the impacts of advanced technology progress for diesel powertrains and conservative technology progress for ZEVs.

Central electricity and hydrogen cost assumptions are similarly based on DOE and NREL estimates. (Note that these values refer to national average costs across all applications. Electricity prices and charging costs will vary significantly across regions, based on time of day and season, station design and utilization and other factors.^41^^,^^46^) Charging costs (inclusive of electricity prices and infrastructure) are taken from a forthcoming NREL analysis which projects the levelized cost of electricity for corridor and depot charging stations until 2050, considering both station installation and maintenance costs and utilization rates.^32^ The share of corridor and depot charging varies with vehicle class and market segment and is further described in Section S3 of the supplemental information. Corridor charging costs begin at $0.40/kWh and decline to $0.24/kWh by 2050 as a result of reduced infrastructure costs and increased utilization. Depot charging costs begin at $0.22/kWh and decline to $0.16 during the same period. These values are similar to the range of prices estimated by Bennett et al.,^40^ who estimated breakeven charging costs ranging from $0.17/kWh to $0.38/kWh for high-power charging of Class 8 trucks (varying with charging speed, electricity rate and utilization). Other studies have identified pathways to achieve charging costs as low as $0.06/kWh,^47^ suggesting a wide range of uncertainty in future MHDV charging costs. To capture some of this uncertainty, our sensitivities include 2050 corridor charging costs ranging from $0.16/kWh to $0.36/kWh, and 2050 depot charging costs ranging from $0.13/kWh to $0.20/kWh. Figure S1.4 documents these cost trajectories. Electric vehicle charging infrastructure is assumed to expand at the rate of the deployment of new EVs, such that lack of infrastructure is not a barrier to adoption. We assume a charging speed of up to 0.5 MW in the central scenario, increasing to up to 1 MW in the most advanced sensitivity for heavy-duty applications.

Hydrogen costs (plotted in Figure S1.5) are assumed to reach $6/kg by 2030 and $4/kg by 2035 under central assumptions and are held constant afterwards, while sensitivities evaluate costs ranging from $7/kg to $3/kg by 2040. These values are in line with those reviewed by Greene et al.,^48^ who compare hydrogen cost projections, inclusive of production, delivery, and station costs across multiple sources. Their review finds levelized costs that range between $4 and $7 by 2030, contingent on capital cost reduction and high-volume production. Similar analysis by the US Hydrogen Interagency Task Force suggests that hydrogen production via electrolysis (not inclusive of delivery and station dispensing costs) can reach $2/kg before 2030 if targets for electrolyzer and balance of plant cost reductions are met and with manufacturing scale-up, in line with a $4/kg total levelized cost target.^33^ However, achieving such targets will require substantial investments and incentives to achieve, which our analysis implicitly includes. Diesel prices (plotted in Figure S1.5) follow the AEO23 central case in the central scenario and average $4/gallon. In the high diesel price scenario, we evaluate the impact of diesel prices that follow the AEO23 *High Oil Price* scenario, an average of $6/gallon.^2^ In the low diesel price scenario, we conversely consider the impact of diesel prices of $2.3/gallon, in line with historical minimums from 2008 to 2019.^36^

The *Central* scenario does not include supportive policies such as vehicle tax credits or sales mandates. We include a sensitivity evaluating the impact of vehicle tax credits aligned with Provision 45W of the IRA. The IRA includes tax credits for qualifying commercial clean vehicles that are the lesser of 30% of the vehicle’s cost, the incremental cost of a ZEV compared to an ICE, or $40,000 for vehicles of 14,000 pounds or more ($7,500 for vehicles below 14,000 pounds).^8^^,^^35^ We model the impact of these credits from 2023 to 2032. We do not represent other provisions of the IRA such as hydrogen, battery, or electricity production tax credits.

Finally, we model a scenario in which range concerns prevent shorter-range EV from becoming available. While our analysis shows that EV-150s (EVs with 150 miles of nominal range) would be the cheapest solutions for several market segments, this scenario assumes a minimum EV range of 300 miles representing a scenario in which vehicle operators find ranges of 150 miles to be insufficient to meet operational needs. This scenario is not meant to imply that such vehicles are technologically infeasible.

In all scenarios, we assume rapid growth in future VMT and ton-miles, based on projections in the 2023 AEO. VMT and ton-miles grow by 33% from 2019 to 2050 across all vehicle classes, with the greatest growth observed in light-medium trucks (increasing by 101% from 2019 to 2050). VMT projections are plotted in Figure S1.6.

## Supplemental information

Document S1. Figures S1.1–S1.6, S2.2, S4.1–S4.3, and S5.1–S5.4, Tables S1.1–S1.4, S2.1, S2.2, S3.1–S3.3, and S4.1, and Section S1–S5
