# Supplementary material for: Assessing total cost of driving competitiveness of zero-emission trucks
Source: iScience. 2024 Mar 2;27(4):109385. doi: 10.1016/j.isci.2024.109385 (PMC10951982; doi:10.1016/j.isci.2024.109385)
Supplement: Document S1. Figures S1.1–S1.6, S2.2, S4.1–S4.3, and S5.1–S5.4, Tables S1.1–S1.4, S2.1, S2.2, S3.1–S3.3, and S4.1, and Section S1–S5 [file mmc1.pdf]

iScience, Volume *27*

Supplemental information

Assessing total cost of driving competitiveness of zero-emission trucks

Catherine Ledna, Matteo Muratori, Arthur Yip, Paige Jadun, Christopher Hoehne, and Kara Podkaminer

Supplemental Information

Section S1. Input Assumptions for Total Cost of Driving, Related to STAR Methods

Table S1.1. Vehicle Activity Assumptions by Class and Shipment Distance Bin. Estimated from on FAF-VIUS Synthesis[1,2], Related to STAR Methods

Vehicle Class	Shipment Distance Bin	Average Annual VMT (Miles)	Average Daily VMT (Miles)*
Light-Medium	0-99 Miles	12,000	<50
	100-249 Miles	56,000	224
	250-499 Miles	26,000	104
	500+ Miles	26,000	104
Medium	0-99 Miles	11,000	<50
	100-249 Miles	64,000	256
	250-499 Miles	60,000	240
	500+ Miles	46,000	184
Heavy	0-99 Miles	<10,000	<50
	100-249 Miles	68,000	272
	250-499 Miles	93,000	372
	500-749 Miles	84,000	280
	750-999 Miles	143,000	477
	1000-1499 Miles	150,000	500
	1500-2000 Miles	153,000	510
	2000+ Miles	206,000	687

* Heavy vehicles in shipment distances of 500+ miles assume 300 operational days per year; all other vehicles assume 250 operational days.

Table S1.2. Detailed Input assumptions for estimating TCD, Related to STAR Methods

Parameter	Definition and Units	Value (*Central* Scenario)	Source
k	Upfront purchase cost (capital cost) of MHDV technology (USD/vehicle)	ICEV and HEV diesel technologies follow *Conservative* technology progress trajectories; ZEV technologies follow *Advanced* trajectory (Figure S1.2)	Islam et al.[3]
fe	Fuel economy of MHDV technology (miles/DGE)	ICEV and HEV diesel technologies follow *Conservative* technology progress trajectories; ZEV technologies follow *Advanced* trajectory (Figure S1.3)	Islam et al.[3]
p	Fuel cost (diesel, hydrogen or electricity) (USD/DGE)	Follows the central trajectories shown in Figure S1.4	Diesel – AEO, Reference and High Oil Price scenarios[4–6]; Electricity charging costs – NREL[7] Hydrogen costs – based on U.S. DOE targets

m	Maintenance cost (USD/mile)	Documented in table S1.3	Hunter et al.[8]
v	Value of EV refueling time (USD/mile)	Described in Section S2; based on opportunity cost of $75/hour from Hunter et al.[8]	
load	Average load factor including dead-heading (tons/vehicle)	Light-medium – 1.2<br>Medium – 2.4<br>Heavy – 10.4	Estimated based on VIUS analysis[2]
vmt	Annual VMT per vehicle (miles/vehicle/year)	Documented in Table S1.1	Estimated based on FAF-VIUS synthesis[1,2]
d	Discount rate used for levelized capital cost computation	7%	
h	Time horizon over which capital costs are levelized	Light-medium – 3 years;<br>Medium – 4 years;<br>Heavy – 5 years	Based on discussion with industry experts
eff	Charging efficiency	90%	Nykvist & Olsson[9]

Table S1.3. Maintenance Cost Assumptions (from Hunter et al.[8]; Mid Scenario), Related to STAR Methods

Vehicle Class	Powertrain	Maintenance Cost (USD/Mile)
Light-Medium and Medium	ICEV and HEV	0.118
	EV	0.076
	FCEV	0.118
Heavy	ICEV and HEV	0.152
	EV	0.098
	FCEV	0.153

Table S1.4. Representative Vehicles by Vehicle Class Used in TEMPO-Autonomie Mapping. Percentages are the weight of each representative vehicle in its respective TEMPO class. Related to STAR Methods.

TEMPO Class	Representative Vehicles from Autonomie[3]
Light-Medium	Class 3 Van (100%)
Medium	Class 4 Parcel & Delivery (41%)<br>Class 6 Box (59%)
Heavy	Class 7 Tractor (4%)<br>Class 8 Tractor (27%)<br>Class 8 Sleeper (69%)

Figure S1.1. Battery Pack and Hydrogen Fuel Cell Price Assumptions Used in Vehicle Cost Modeling from Islam et al.[3]. The central scenario assumes advanced technology progress assumptions, with conservative progress explored in sensitivities. Related to STAR Methods

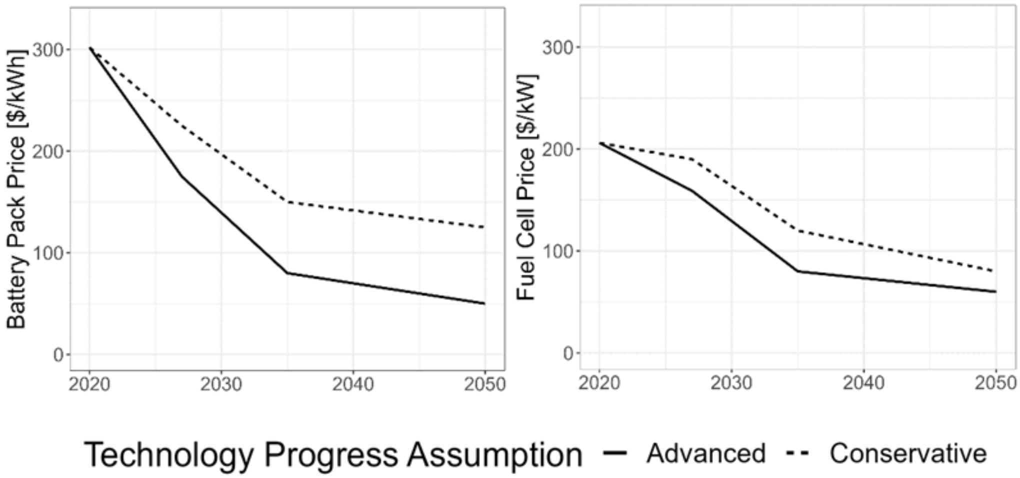

Figure S1.2. TEMPO Vehicle Capital Cost Inputs, Aggregated to TEMPO Resolution from Islam et al.[3]
Related to STAR Methods

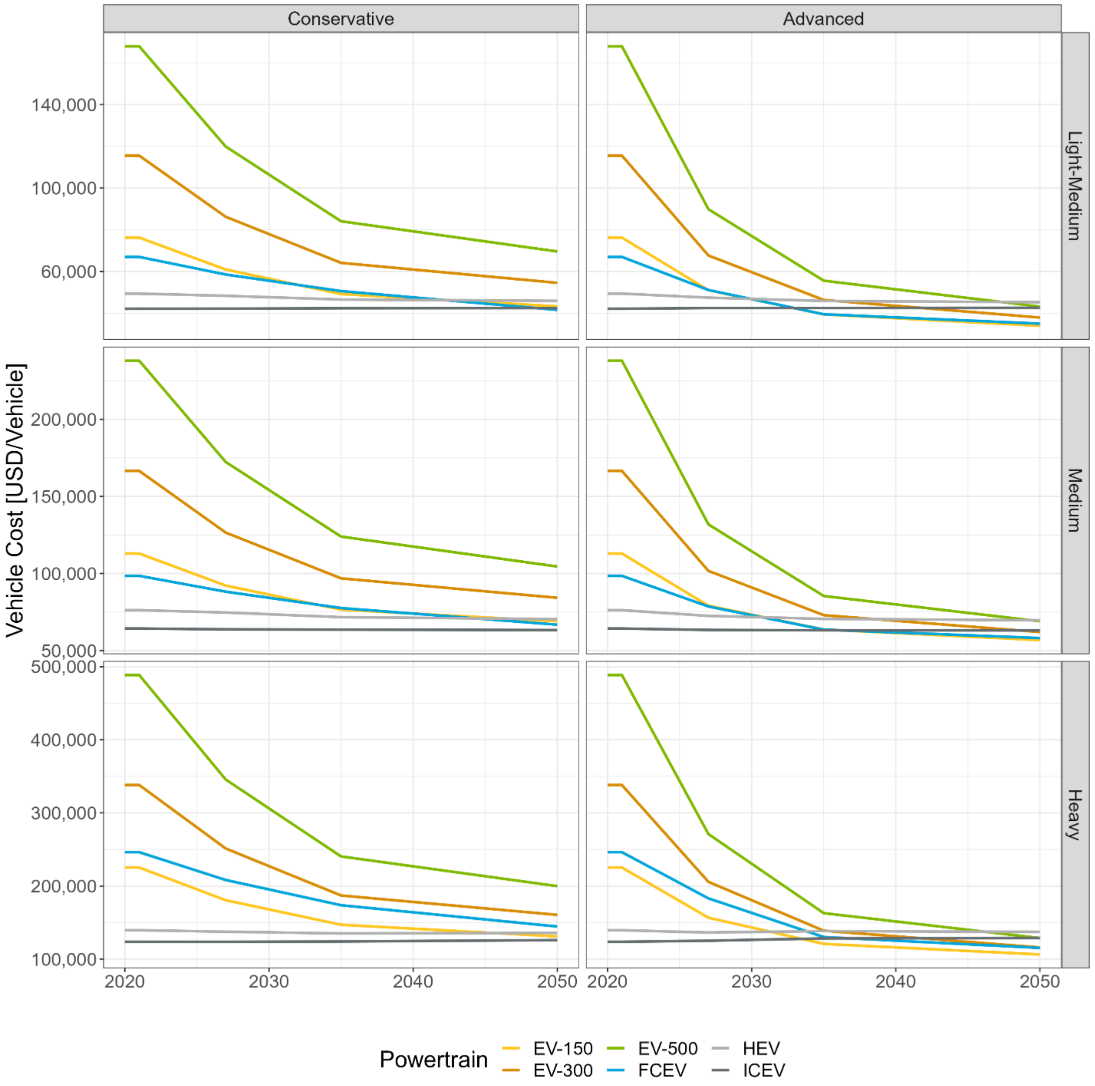

Figure S1.3. TEMPO Vehicle Fuel Economy Inputs in Miles Per Diesel Gallon Equivalent (DGE), Aggregated from Islam et al.[3] Related to STAR Methods

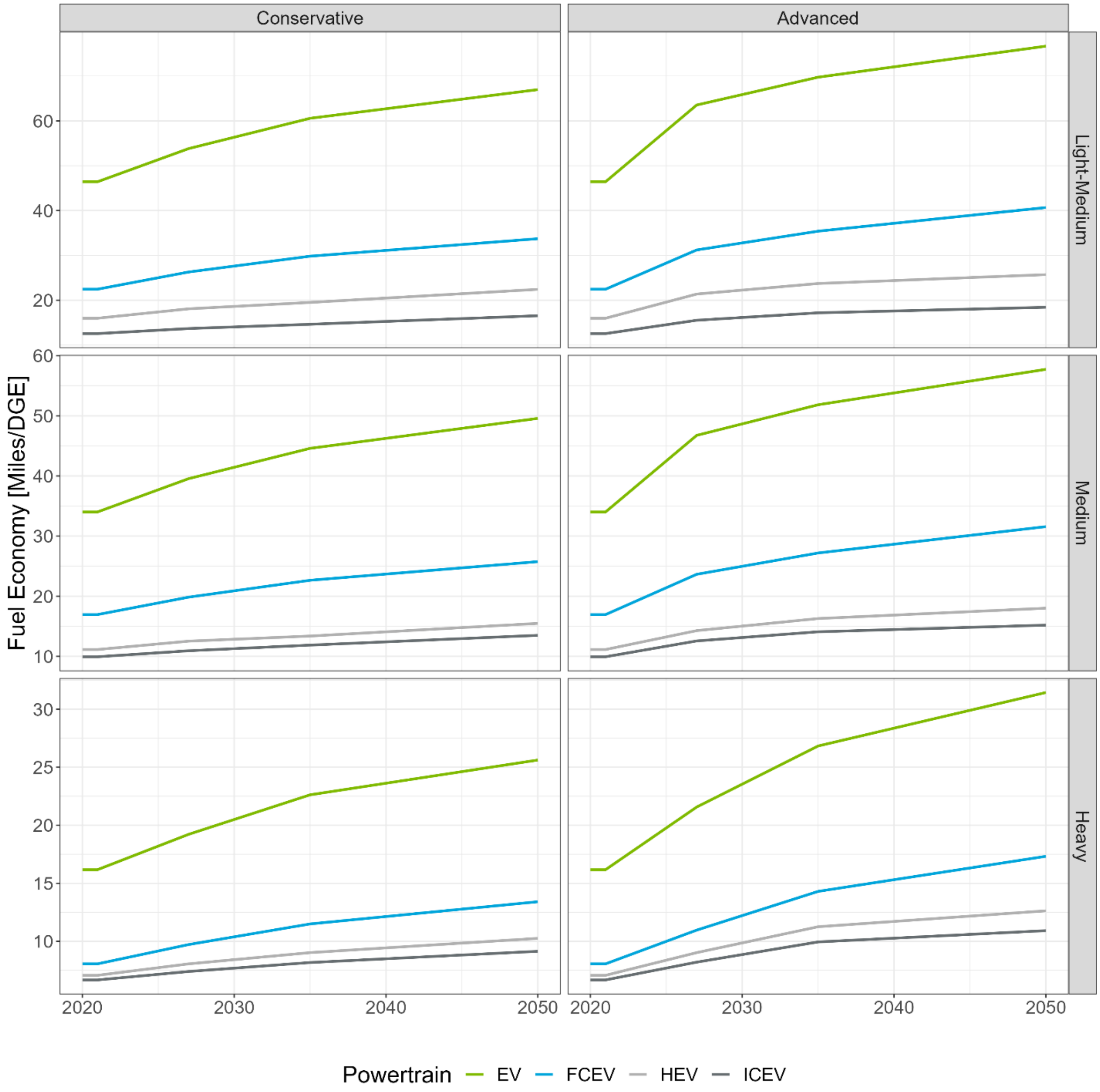

Figure S1.4. Charging Cost Trajectories, *Central* Scenario and Sensitivities. Based on forthcoming NREL analysis.[7] Costs include the levelized cost of charging infrastructure. Related to Table 2 and STAR Methods.

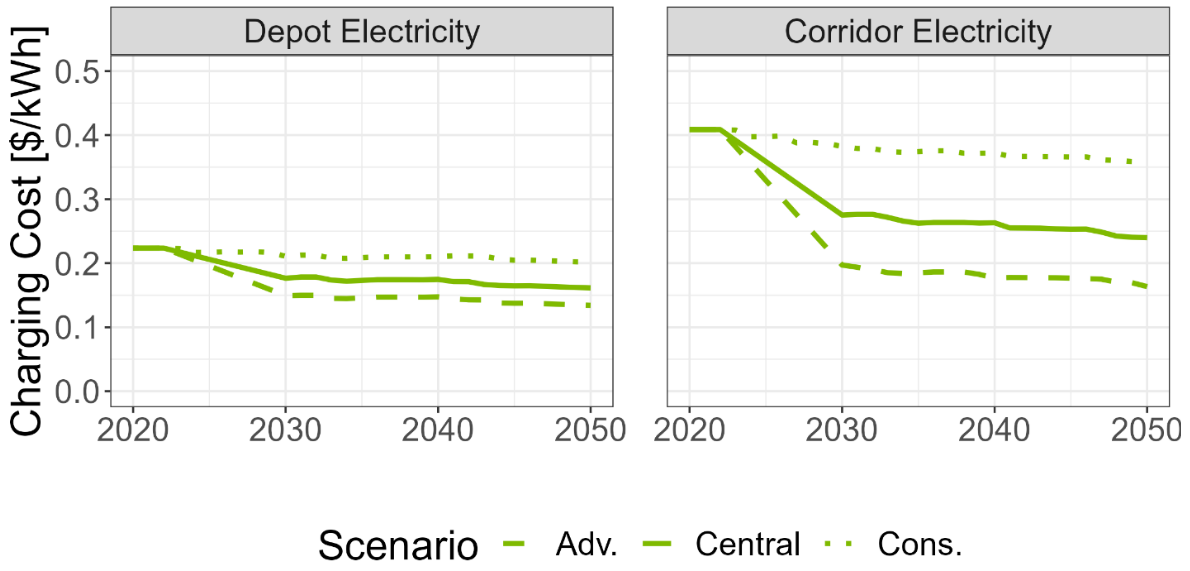

Figure S1.5. Hydrogen and Diesel Cost Trajectories, *Central* Scenario and Sensitivities. Central and high diesel costs from EIA AEO[4–6]; low diesel costs from EIA historical data[10] Hydrogen costs based on U.S. DOE targets and include refueling infrastructure.[11] Related to Table 2 and STAR Methods.

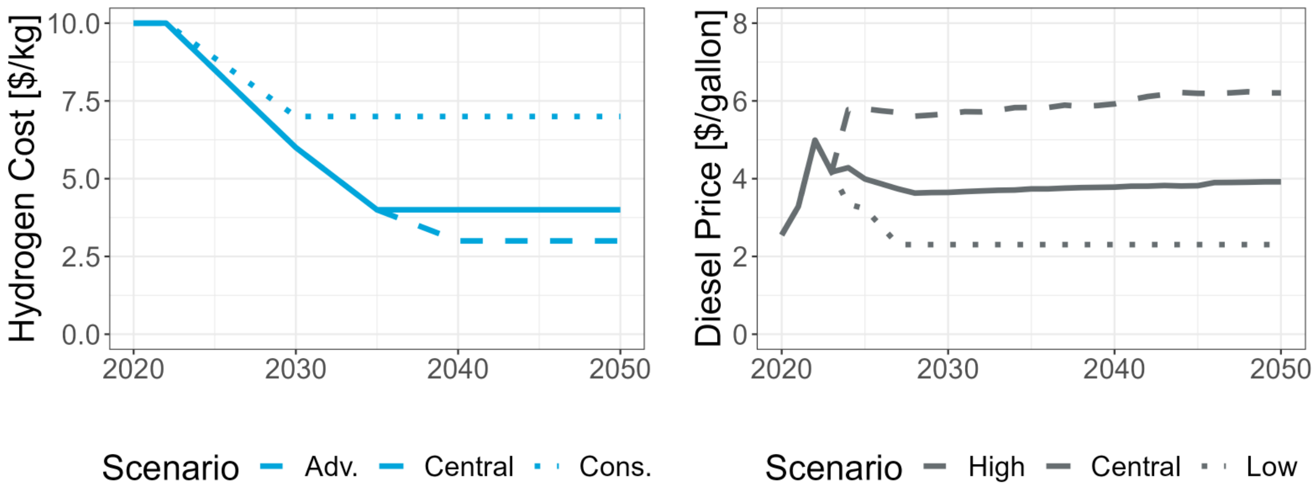

Figure S1.6. VMT Projections by Vehicle Class. Based on EIA AEO trajectories.[4–6] Related to STAR Methods.

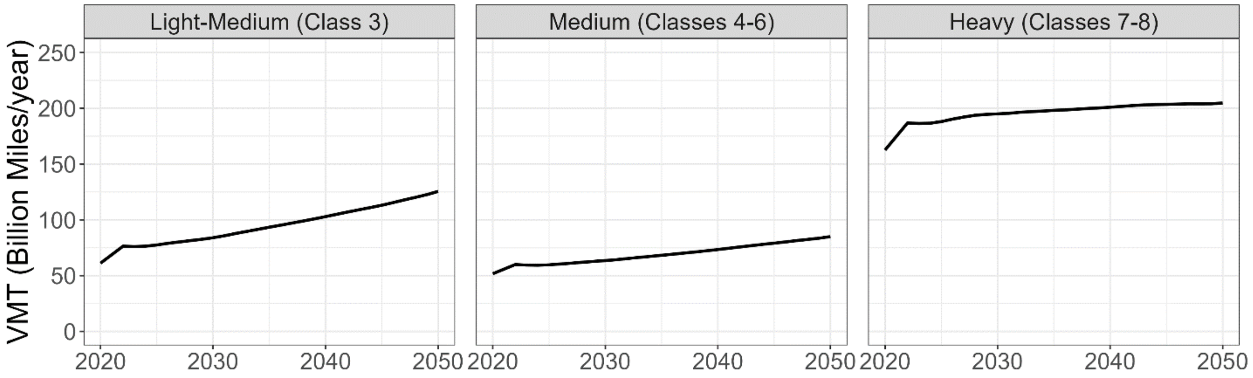

Section S2. Market Segmentation, Related to STAR Methods.

Table S2.1. Mapping of FAF Shipment Distance to VIUS Primary Operating Distance. Related to STAR Methods.

FAF Shipment Distance	VIUS Primary Operating Distance
0—99 Miles	Less than 50 miles, 51-100 miles
100—249 Miles	101-200 miles
250—499 Miles	201-500 miles
500—749 Miles	501 or more miles
750—999 Miles	501 or more miles
1000—1499 Miles	501 or more miles
1500—1999 Miles	501 or more miles
2000+ Miles	501 or more miles

Table S2.2. MHDV Freight and Non-Freight Stock and VMT Shares by Vehicle Class. Based on data provided by NREL.[12] Related to STAR Methods.

Vehicle Class	Vehicle Use	Stock Share (%)	VMT Share (%)
Light-Medium (Class 3)	Freight	31	24
	Non-Freight	69	76
Medium (Class 4-6)	Freight	58	61
	Non-Freight	42	39
Heavy (Class 7-8)	Freight	81	94
	Non-Freight	19	6

Figure S2.2. Comparison of TEMPO and VIUS Distribution of Annual VMT by Class. Reproduced from Ledna et al.[13] Related to STAR Methods.

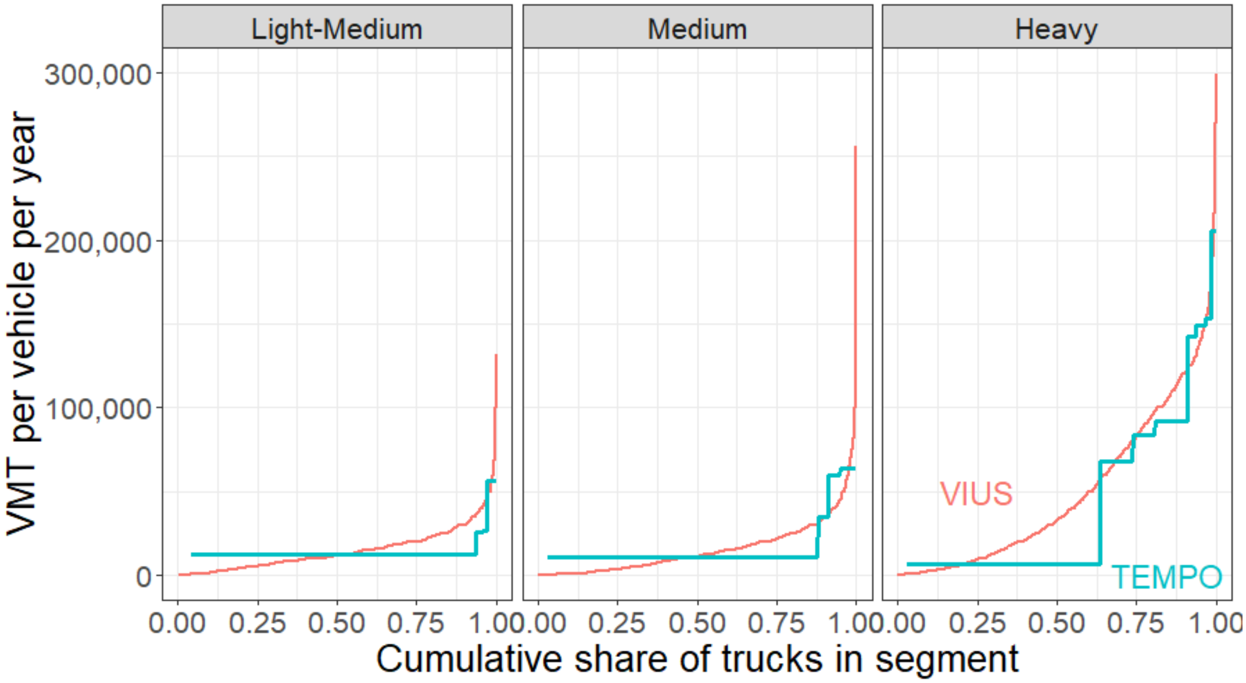

Section S3. Methods for Estimating EV Charging Cost and Opportunity Cost of EV Recharging Time. Related to STAR Methods.

To estimate fuel and charging time costs for EVs, we estimate the shares of EV charging that occur during en-route and opportunity dwells. We define en-route charging as charging that would interrupt the EV's daily operations. Opportunity charging is defined as charging that would occur when the vehicle would otherwise be inactive (e.g., when charging overnight). En-route charging is assumed to use corridor charging, while opportunity charging is assumed to use depot charging (charging costs are plotted in Figure S1.4). En-route charging carries an additional opportunity cost based on the time spent recharging when the vehicle would otherwise be driving. The miles attained through opportunity charging have no opportunity cost.

We first estimate the share of energy consumed during en-route charging for EVs by class and market segment. We use Equation S3.1 to estimate the share of energy consumed from en-route charging ($frac_{enroute,c,t,s}$) for vehicle class *c,* technology *t,* and market segment *s.*

$$frac_{enroute,c,t,s} = \frac{(dailyvmt_{c,s} - range_t \times (1 - minsoc)) \times operatingdays_{c,s}}{annualvmt_{c,s}} \times \left(1 - frac_{continuous,c,s}\right) + frac_{continuous,c,s}$$

(Equation S3.1)

The fraction of en-route charging needed for each market segment is estimated as a weighted average of continuously operating vehicles ($frac_{continuous,c,s}$), who use only en-route charging, and non-continuously operating vehicles, who have access to opportunity charging and may use only some en-route charging. We assume that non-continuously operating vehicles have ubiquitous access to overnight or opportunity depot charging and begin each day fully charged. For these vehicles, the en-route charging share is estimated as the share of daily VMT that exceeds the maximum allowable range, multiplied by the number

of operating days and divided by total annual VMT. The values $dailyvmt_{c,s}$ and $annualvmt_{c,s}$ are the average daily and annual VMT for each class and market segment (listed in Table S1.1), $range_t$ is the EV's range in miles, and $minsoc$ is the minimum state of charge the battery can reach before requiring recharging (assumed to be 0.1 for all EVs, consistent with Borlaug et al.[14]). This implies that if the vehicle's daily mileage is less than 90% of the vehicle range, they can complete their daily operations without any en-route charging. Vehicles are assumed to operate either 250 or 300 days per year ($operatingdays_{c,s}$), varying as defined in Table S1.1. The fraction of continuously operating vehicles ($frac_{continuous,c,s}$) is taken from Schoettle et al.[15], based on their estimates of the fraction of team driving operations in heavy-duty market segments and is listed in Table S3.1.

Table S3.1. Team Driving Assumptions by Vehicle Class and Market Segment. Based on Schoettle et al.[15] Related to STAR Methods.

Vehicle Class	Market Segment	Team Driving (Share of Vehicles)
Light-Medium & Medium	All	0%
Heavy	0-99 Miles to 250-499 Miles	0%
	500+ Miles	38%

For some heavy-duty vehicle market segments (classes 7-8), we leverage data from Borlaug et al.[14], who estimate en-route and opportunity charging shares for local, regional, and long-haul operations based on real-world operational data, rather than using Equation S3.1. We map Borlaug et al.'s estimates to TEMPO's heavy-duty operational classes based on average daily and annual VMT. The '100-249 Mile' market segment is mapped to Borlaug et al.'s 'Local' segment, the '250-499' and '500-749' mile market segments are mapped to the 'Regional' segment, and the '750-999' mile market segment is mapped to the 'Long-Haul' segment. Other TEMPO market segments (0-99 miles and 1000+ miles) were found to have substantially different daily and annual mileage than the representative vehicle operations in Borlaug et al. and were instead estimated using Equation S3.1. Our final shares of en-route and opportunity charging are listed in Table S3.2.

Table S3.2. En-Route and Opportunity Charging Shares by EV Class, Range and Market Segment. Related to STAR Methods.

<table>
<tr><th>Vehicle Class</th><th>Electric Range (Miles)</th><th>Market Segment</th><th>En-Route Charging Share (Corridor)</th><th>Opportunity Charging Share (Depot)</th></tr>
<tr><td rowspan="4">Light-Medium (Class 3)</td><td rowspan="2">150</td><td>0-99 Miles; 250-499 Miles; 500+ Miles</td><td>0%</td><td>100%</td></tr>
<tr><td>100-249 Miles</td><td>40.1%</td><td>59.9%</td></tr>
<tr><td>300</td><td>All segments</td><td>0%</td><td>100%</td></tr>
<tr><td>500</td><td>All segments</td><td>0%</td><td>100%</td></tr>
<tr><td rowspan="6">Medium (Classes 4-6)</td><td rowspan="4">150</td><td>0-99 Miles</td><td>0%</td><td>100%</td></tr>
<tr><td>100-249 Miles</td><td>47%</td><td>53%</td></tr>
<tr><td>250-499 Miles</td><td>43.5%</td><td>56.5%</td></tr>
<tr><td>500+ Miles</td><td>26.4%</td><td>73.6%</td></tr>
<tr><td>300</td><td>All segments</td><td>0%</td><td>100%</td></tr>
<tr><td>500</td><td>All segments</td><td>0%</td><td>100%</td></tr>
<tr><td rowspan="7">Heavy (Classes 7-8)</td><td rowspan="7">150</td><td>0-99 Miles</td><td>0%</td><td>100%</td></tr>
<tr><td>100-249 Miles</td><td>54%</td><td>46%</td></tr>
<tr><td>250-499 Miles</td><td>71%</td><td>29%</td></tr>
<tr><td>500-749 Miles</td><td>71%</td><td>29%</td></tr>
<tr><td>750-999 Miles</td><td>76%</td><td>24%</td></tr>
<tr><td>1000-1499 Miles</td><td>83.3%</td><td>16.7%</td></tr>
<tr><td>1500-1999 Miles</td><td>83.7%</td><td>16.3%</td></tr>
</table>

		2000+ Miles	87.9%	12.1%
	300	0-99 Miles	0%	100%
		100-249 Miles	26%	74%
		250-499 Miles	46%	54%
		500-749 Miles	46%	54%
		750-999 Miles	55%	45%
		1000-1499 Miles	66.7%	33.3%
		1500-1999 Miles	67.4%	32.6%
		2000+ Miles	75.8%	24.2%
	500	0-99 Miles	0%	100%
		100-249 Miles	11%	89%
		250-499 Miles	23%	77%
		500-749 Miles	23%	77%
		750-999 Miles	33%	67%
		1000-1499 Miles	44.5%	55.5%
		1500-1999 Miles	45.7%	54.3%
		2000+ Miles	56.9%	43.1%

The opportunity cost of en-route charging, *v,* in USD per mile for vehicle class *c,* technology *t*, market segment *s* and year *y* is computed as follows:

$$v_{c,t,s,y} = j \times r_{c,t,y} \times frac_{enroute,c,t,s} \text{ (Equation S3.2)}$$

Where *j* is the cost in USD per hour of time spent recharging (assumed to be $75/hour, based on Hunter et al.[8]), *r* is the charging time in hours per mile (a function of battery size and charging power), and $frac_{enroute,c,t,s}$ is listed in Table S3.2. Charging time is computed as follows:

$$r_{c,t,y} = \frac{fe_{c,t,y}}{charging\_speed} \text{ (Equation S3.3)}$$

Where *fe* is the fuel economy in kWh per mile and *charging_speed* is the charging speed in kW. Charging speed assumptions are listed in Table S3.3 and vary by scenario. In all scenarios, we limit maximum charging speed to 350 kW for light-medium vehicles and 500 kW for medium and short-range heavy vehicles to account for limitations based on battery capacity, temperature and C-rate.[16] Heavy EVs with 300 miles of range or greater are assumed to be able to charge at up to 1 MW speeds.

Table S3.3. Charging Speed Assumptions by EV Class, Technology and Scenario

Vehicle Class	EV Range	Charging Speed (kW), *Adv. Electricity* scenario	Charging Speed (kW), *Central* and all other scenarios
Light-Medium (Class 3)	150	350	350
	300	350	350
	500	350	350
Medium (Classes 4-6)	150	500	500
	300	500	500
	500	500	500
Heavy (Classes 7-8)	150	500	500
	300	1000	500
	500	1000	500

Section S4. Vehicle Stock Model and Model Validation. Related to STAR Methods.

We model annual MHDV stock turnover, including new vehicle sales, the population and activity of existing vehicles, and vehicle retirements using the TEMPO model. Stock turnover is modeled in TEMPO using the following steps:

1. Estimate annual freight demand for each vehicle class in ton-miles. Initial (2017) ton-miles are based on the Freight Analysis Framework (FAF)[1], and grow at the rate of annual projected VMT growth in the Annual Energy Outlook (AEO).[4–6]
2. Estimate freight demand in ton-miles met by existing vehicle stock using annual VMT per vehicle and load factor (tons per vehicle) assumptions. Average annual VMT per vehicle assumptions are documented in Table S1.1.
3. Compute the gap between existing and demanded ton-miles. If demand for ton-miles is greater than what the existing fleet can supply, new vehicles are added to the fleet using a ton-miles per vehicle conversion factor. If demand is less than what existing vehicles can supply, existing vehicles are driven less to model fewer shipments.
4. Retire vehicles from the fleet based on age-specific survival probabilities.

The initial (2017) US fleet age distribution is based on MOVES.[17] Age-specific survival probabilities are based on survival curves provided by the Energy Information Administration.[18] Cumulative survival probability by vehicle age is plotted in Figure S4.1. Vehicles are assumed to fully exit the fleet after age 34. No difference in survival was assumed between vehicle technologies or operating segments within a class. The initial age distribution and survival curves result in an average vehicle lifetime of 20 years for Class 3, 19 years for Classes 4-6, and 21 years for Classes 7-8, and an average fleet age of 12.6 years for Class 3, 12 years for Classes 4-6, and 13.4 years for Classes 7-8.

Figure S4.1. Cumulative Vehicle Survival Probability by Age and Vehicle Class. Based on scrappage rates provided by the Energy Information Administration.[18] Related to STAR Methods.

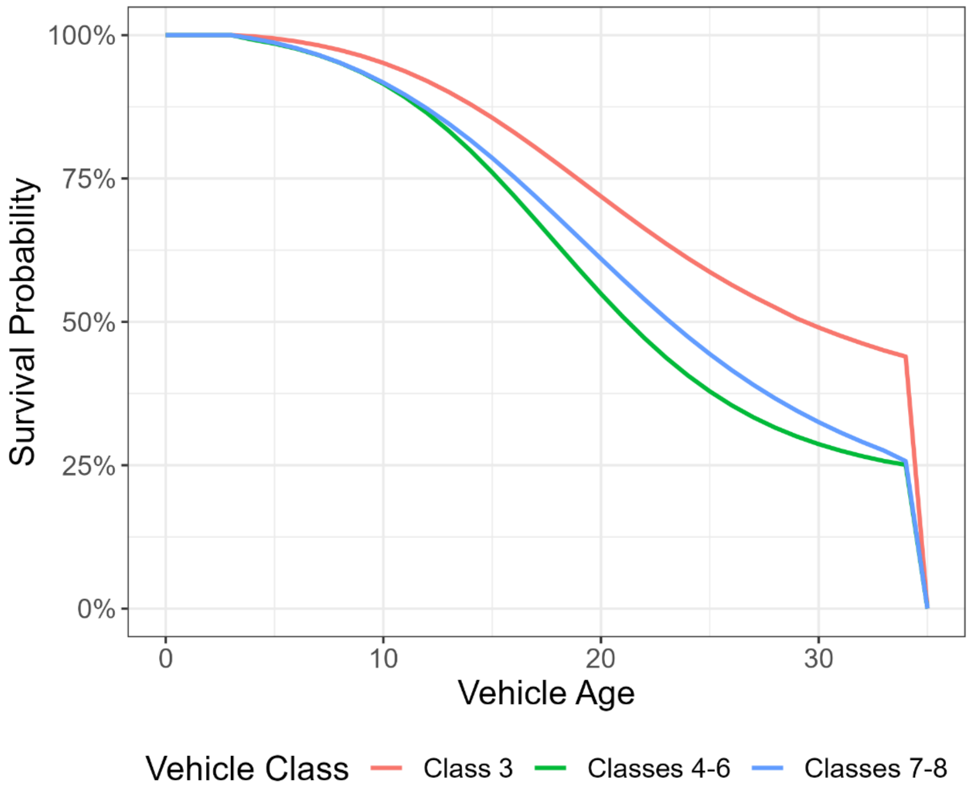

To validate TEMPO's projections of total MHDV stock and sales, we compare total MHDV stock (Figure S4.2) and sales (Figure S4.3) to projections from the AEO. Modeled vehicle stock in TEMPO closely matches AEO projections, with slight differences arising from differences in vehicle load-factors. The largest differences in stock are for light-medium vehicles, which are 9% lower in 2050 compared to AEO projections. Vehicle sales fluctuations in TEMPO in years prior to 2023 due to the impact of demand changes during the COVID-19 pandemic, but closely match AEO in later years. We also compare modeled energy consumption and GHG emissions to 2019 values (Table S4.1); our results are within 2% of historical values in 2019.

Figure S4.2. Vehicle Stock Comparison, TEMPO and AEO[6]. Related to STAR Methods.

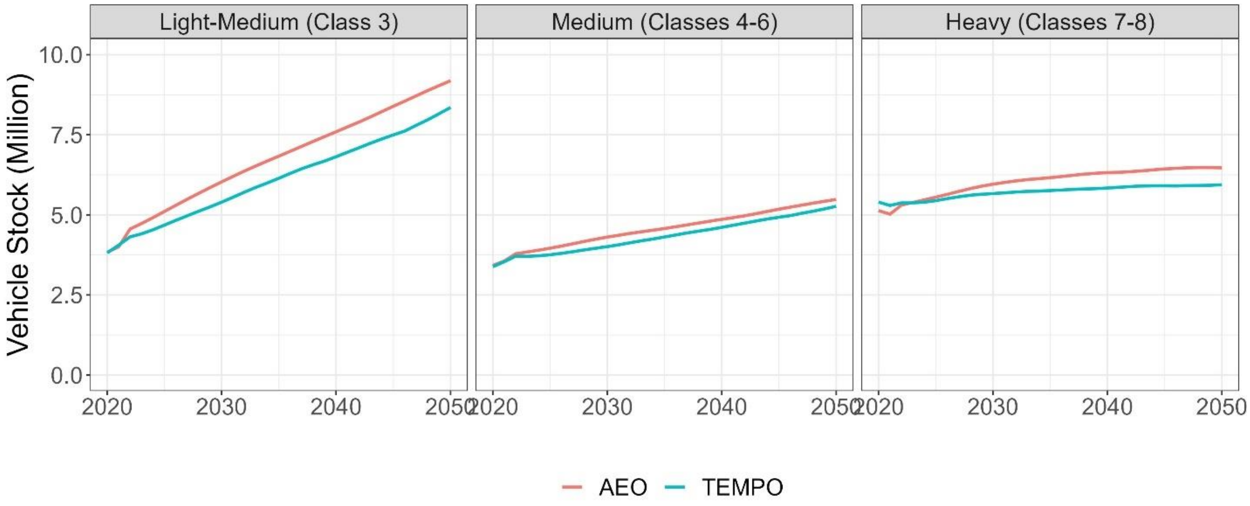

Figure S4.3. Vehicle Sales Comparison, TEMPO (3-year average) and AEO[6]. Related to STAR Methods.

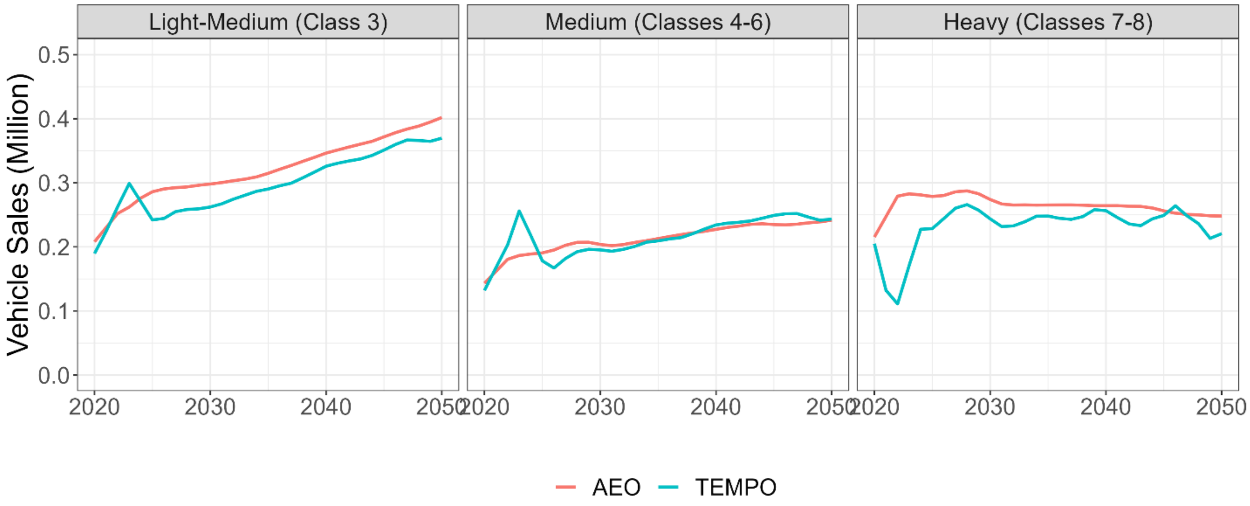

Table S4.1. Modeled 2019 Energy Consumption and GHG Emissions Compared to Historical Values. Related to STAR Methods.

	Historical, 2019*	TEMPO, 2019
Energy Consumption (Quads)	5.7	5.8
GHG Emissions (MMT CO_2e)	439.5	431

*Based on U.S. Department of Energy compiled values.[19] May differ from other sources due to differences in definitions or scope.

Section S5. Supplemental Results

Figure S5.1 Total Cost of Driving, *Central* Scenario, Selected Classes and Market Segments, 2025-2050. Related to Figure 2.

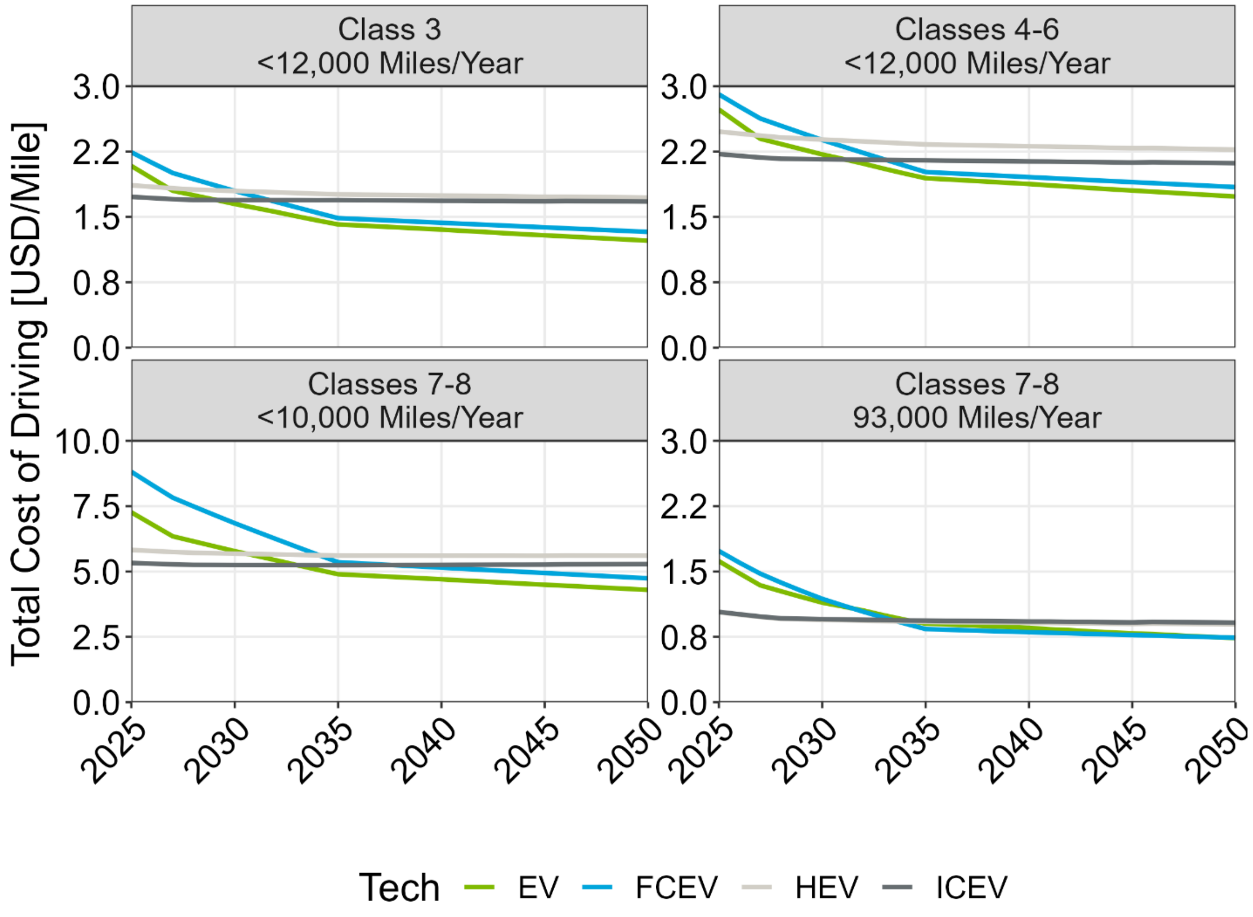

Figure S5.2. Sensitivity of Heavy (Class 7-8) Trucks to Altered Fuel Cost Assumptions, 2035, Central Technology Progress Assumptions and 0.5 MW Charging Speed. Related to Figure 7.

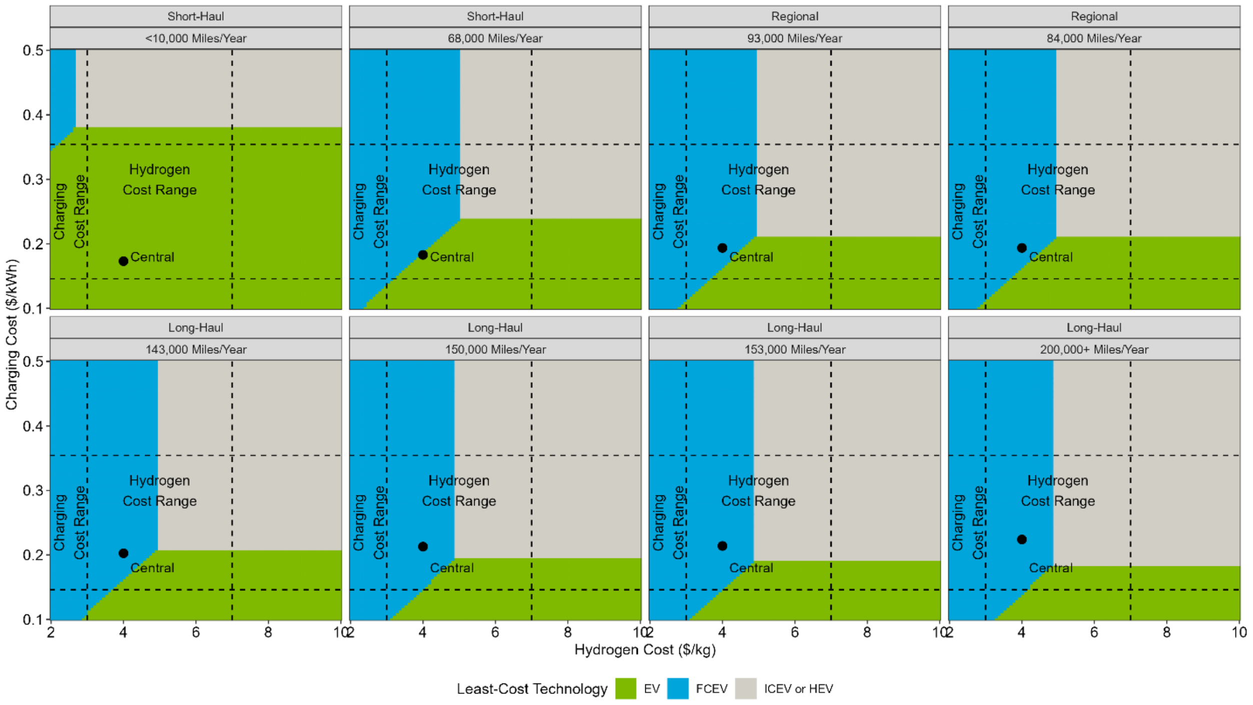

Figure S5.3. Sensitivity of Heavy (Class 7-8) Trucks to Altered Fuel Cost Assumptions, 2035, Central Technology Progress Assumptions and 1 MW Charging Speed. Related to Figure 7.

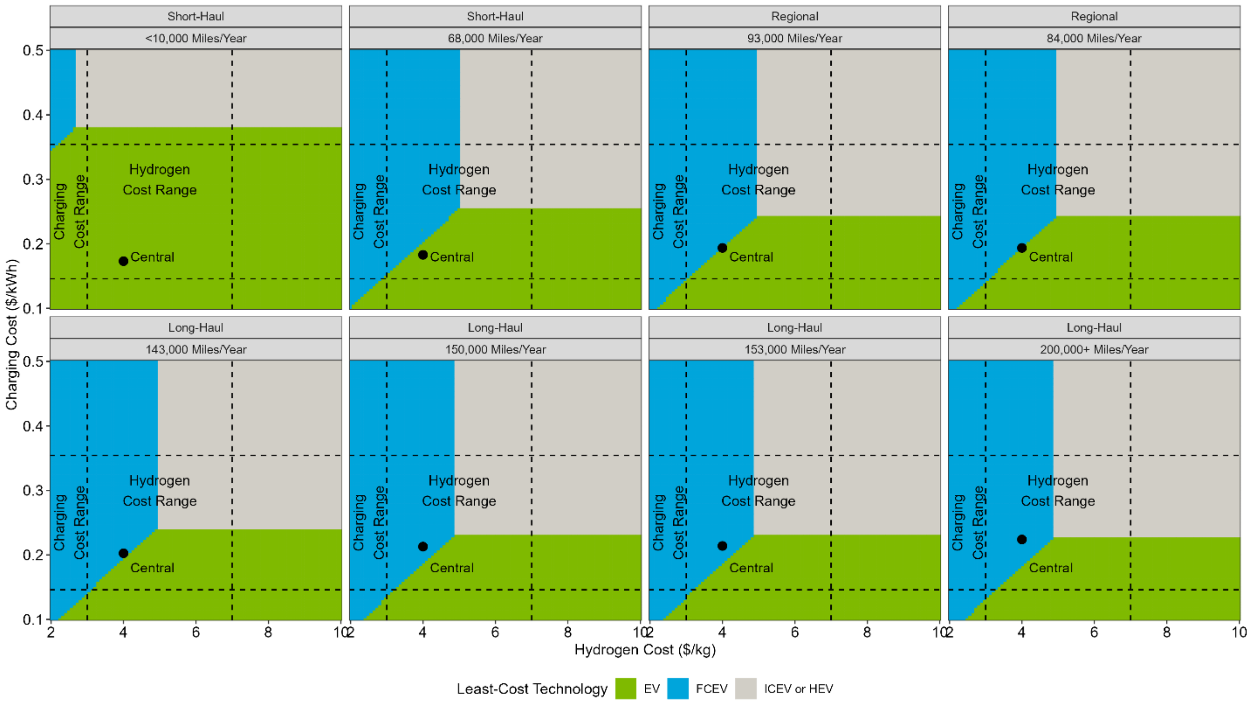

Figure S5.4. Sensitivity of Heavy (Class 7-8) Trucks to Altered Fuel Cost Assumptions, 2050, Central Technology Progress Assumptions and 0.5 or 1 MW Charging Speed. Related to Figure 7.

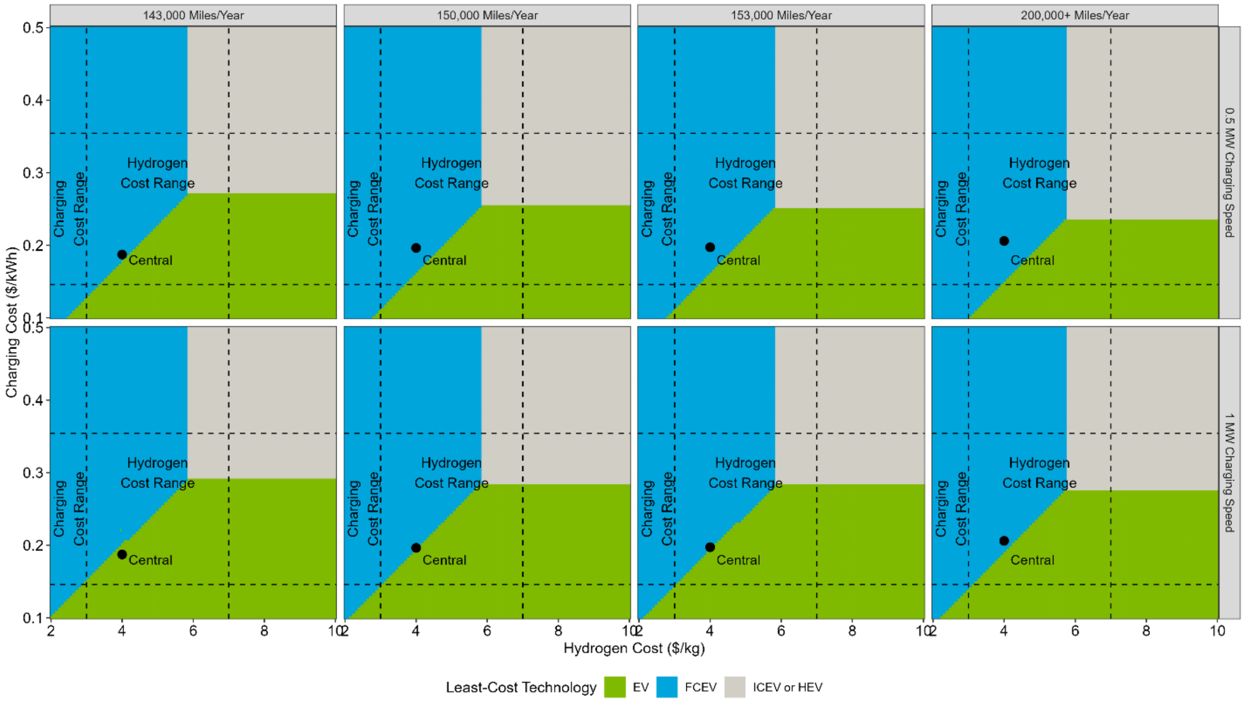

References

1. Federal Highway Administration (2019). Freight Analysis Framework - 2017 (Federal Highway Administration).

2. U.S. Census Bureau (2004). Vehicle Inventory and Use Survey (VIUS). https://www.census.gov/library/publications/2002/econ/census/vehicle-inventory-and-use-survey.html.

3. Islam, E.S., Vijayagopal, R., Moawad, A., Kim, N., Dupont, B., Nieto Prada, D., and Rousseau, A. (2021). A Detailed Vehicle Modeling & Simulation Study Quantifying Energy Consumption and Cost Reduction of Advanced Vehicle Technologies Through 2050 (Argonne National Laboratory).

4. EIA (2019). Annual Energy Outlook 2019 (U.S. Energy Information Administration).

5. EIA (2021). Annual Energy Outlook 2021 with projections to 2050 (Energy Information Administration, U.S. Department of Energy).

6. EIA (2023). Annual Energy Outlook 2023 (U.S. Energy Information Administration).

7. NREL (Forthcoming). MHDV Corridor and Depot Charging Costs (National Renewable Energy Laboratory).

8. Hunter, C., Penev, M., Reznicek, E., Lustbader, J., Birky, A., and Zhang, C. (2021). Spatial and Temporal Analysis of the Total Cost of Ownership for Class 8 Tractors and Class 4 Parcel Delivery Trucks (National Renewable Energy Laboratory).

9. Nykvist, B., and Olsson, O. (2021). The feasibility of heavy battery electric trucks. Joule *5*, 901–913. https://doi.org/10.1016/j.joule.2021.03.007.

10. EIA (2023). U.S. No 2 Diesel Ultra Low Sulfur (0-15 ppm) Retail Prices (U.S. Energy Information Administration).

11. US Hydrogen Interagency Task Force (2023). US National Clean Hydrogen Strategy and Roadmap (US Hydrogen Interagency Task Force).

12. National Renewable Energy Laboratory Data provided by NREL based on analysis of 2013 IHS Polk vehicle registrations, the 2002 Vehicle Inventory and Use Survey, 2018 data from the American Public Transportation Association, Federal Highway Administration data, and other sources.

13. Ledna, C., Muratori, M., Yip, A., Jadun, P., and Hoehne, C. (2022). Decarbonizing Medium- & Heavy-Duty On-Road Vehicles: Zero-Emission Vehicles Cost Analysis (National Renewable Energy Laboratory).

14. Borlaug, B., Moniot, M., Birky, A., Alexander, M., and Muratori, M. (2022). Charging needs for electric semi-trailer trucks. Renewable and Sustainable Energy Transition *2*, 100038.

15. Schoettle, B., Sivak, M., and Tunnell, M. (2016). A survey of fuel economy and fuel usage by heavy-duty truck fleets (University of Michigan Sustainable World Transportation & American Transportation Research Institute).

16. Pradhan, R., Keshmiri, N., and Emadi, A. (2023). On-Board Chargers for High-Voltage Electric Vehicle Powertrains: Future Trends and Challenges. IEEE Open Journal of Power Electronics *4*, 189–207.

17. EPA (2021). Population and Activity of Onroad Vehicles in MOVES3 (U.S. Environmental Protection Agency).

18. EIA (2022). MHDV Scrappage Rates.

19. DOE, DOT, EPA, and HUD (2023). The U.S. National Blueprint for Transportation Decarbonization (U.S. Department of Energy, U.S. Department of Transportation, U.S. Environmental Protection Agency, and U.S. Department of Housing and Urban Development).
